# Non-Arrhenius threshold switching by field-driven dipolar ordering

**DOI:** 10.1038/s41467-026-72970-z

**Published:** 2026-05-13

**Authors:** Wen-Xiong Song, Guangjie Shi, Qi Hu, Fan Zhu, Tianjiao Xin, Ying Chen, Sergiu Clima, Gilberto Teobaldi, Yuhao Wang, Wenjian Huang, Sannian Song, Cheol Seong Hwang, Li-Min Liu, Yan Cheng, Zhitang Song

**Affiliations:** 1 State Key Laboratory of Materials for Integrated Circuits, Shanghai Institute of Microsystem and Information Technology, Chinese Academy of Sciences, Shanghai, China; 2 Key Laboratory of Polar Materials and Devices (MOE), School of Information and Electronic Engineering & School of Integrated Circuits Science and Engineering, East China Normal University, Shanghai, China; 3 School of Physics, Beihang University, Beijing, China; 4 Department of Materials Science, Fudan University, Shanghai, China; 5 Key Laboratory of Inorganic Functional Materials and Devices, Shanghai Institute of Ceramics, Chinese Academy of Sciences, Shanghai, China; 6 imec, Kapeldreef 75, Leuven, Belgium; 7 Scientific Computing Department, STFC UKRI, Rutherford Appleton Laboratory, Didcot, UK; 8 Department of Materials Science and Engineering and Inter-University Semiconductor Research Center, Seoul National University, Seoul, South Korea

**Keywords:** Information storage, Structure of solids and liquids, Electronics, photonics and device physics

## Abstract

The long-standing challenge in resolving the atomic-scale threshold switching mechanism in amorphous chalcogenides, fundamental constraint on further development of promising memory technologies, stems from their intrinsic structural disorder. Here, we overcome this pivotal challenge by capturing electric-field-driven dipolar ordering in amorphous GeSe through combined atomic-resolution angstrom-beam electron diffraction and field-coupled ab initio molecular dynamics. Electric fields induce anti-parallel displacements of Ge ( + 0.23 Å) and Se ( − 0.21 Å) atoms within picoseconds, aligning dipoles into one-dimension chains. These polarity-locked chains, evidenced by two distinct diffraction spots (1.95 Å spacing), guide conductive filament growth perpendicular to chain alignment. This mechanism enables direct harnessing of dipole-originated threshold voltage asymmetry in selector-only memory, achieving dual functionality through single-material engineering. This field-induced non-Arrhenius process squashes thermal activation barriers, enabling dipolar-order-driven switching within the picosecond regime thus breaking the thermal speed limit for resistive switching. Our findings establish a pathway to atomic-scale dipole control for ultrafast storage-class memory.

## Abstract (web summary)

Amorphous chalcogenides enable ultrafast threshold switching, yet their switching mechanism remains debated between electronic and structural models. Song et al. reveal an electric field induced atomic displacement and dipolar ordering, which exhibit a non-Arrhenius structural transition and guide conductive filament formation.

## Introduction

The incoherent motion of atoms in amorphous materials, long viewed as a barrier to functionality, can paradoxically enable ultrafast responses to external stimuli. Deciphering how atomic-scale disorder orchestrates mesoscale order holds the key to quantitatively understanding out-of-equilibrium matter, with far-reaching implications spanning brain-inspired computing to energy-efficient chalcogenide-based memories. Amorphous chalcogenides, first reported in the 1960s for their abrupt (>3 orders of magnitude) conductivity jumps at a critical threshold switching voltage (*V*_th_)^1^, epitomize this complexity. These materials have revolutionized non-volatile memory technologies by shattering the “memory wall” bottleneck of conventional architectures through ultralow-latency operation, a critical enabler for current artificial intelligence^2,3^ and big data^4^ applications that demand high-throughput data processing. While phase-change random access memory uses amorphous chalcogenide ovonic threshold switching (OTS) as a selector element and ovonic memory switching (OMS) as a memory element^5^, the newer selector-only memory (SOM)^6–10^, termed self-rectifying OTS memory (SR-OTSM), self-selecting memory (SSM), or single-chalcogenide Xpoint memory (SXM), eliminates the OMS layer and its slow crystallization, achieving faster switching speed and lower power consumption.

Despite five decades of research^11–25^, the atomic-scale mechanism of threshold switching remains elusive. While substantial insights have emerged from two dominant frameworks, electronic-driven models^11–13,16–19,22–25^ and structural transition-based models^14,15^, critical gaps persist. Electronic models successfully describe conductivity while treating atomic relaxation as decoupled. For example, the avalanche model^25^ attributes the threshold phenomenon to impact ionization of gap states originating from valence alternation pairs (VAPs)^13^, where carrier multiplication triggers an electronic avalanche^25^. Conversely, the structural transition-based model attributes the switching to the nucleation and growth of conductive filaments (CFs), conceptualized as nanoscale crystallite-like ordered domains, via field-induced structural rearrangements^14,15^. It successfully describes the drift dynamics of *V*_th_ value, yet lacks explicit connections to electronic properties and does not account for sub-picosecond CF formation under THz fields^16^, where 10^3^–10^6^× acceleration exceeds the predictions of thermally activated nucleation processes by orders of magnitude. Therefore, a unified structural-electronic mechanism is a prerequisite to accelerate the rational design of high-performance materials, enabling breakthroughs in non-volatile memory technologies.

Here, we establish a picosecond-scale dipolar quantum ordering paradigm, where field-induced atomic displacements directly govern ultrafast electronic transitions in amorphous networks. We combine angstrom-beam electron diffraction (ABED: 6.56 Å electron beam) and first principles, field-coupled molecular dynamics (EF-MD) to resolve dipolar reconfiguration dynamics, directly mapping field-aligned motifs in amorphous matrices. This multimodal synergy bridges atomic-scale dipolar signatures to mesoscale conductive pathways, a leap unattainable by prior static or field-free characterization. The OFF-to-ON transition is driven by a non-Arrhenius process, because the field-driven dipolar alignment of Ge–Se bonds triggers picosecond-scale structural ordering of amorphous motifs, overcoming energy barriers at a rate three orders of magnitude faster than thermal nucleation kinetics. This accelerated dynamics originates from the synergistic effect of the high electric field and the enhanced dipole moment, which alter the potential energy landscape governing the system’s nuclear dynamics. Then, following the generation of CF percolation via ordered motifs at the mesoscale, threshold switching of the device is eventually achieved. This hierarchy, bridging atomic dipolar dynamics to macroscopic functionality, redefines chalcogenide memory design based on dipole stability criteria and introduces new paradigms for harnessing disorder in quantum materials.

## Results and discussion

### Conductive filament formation via dipolar chain alignment

**Fig. 1:**
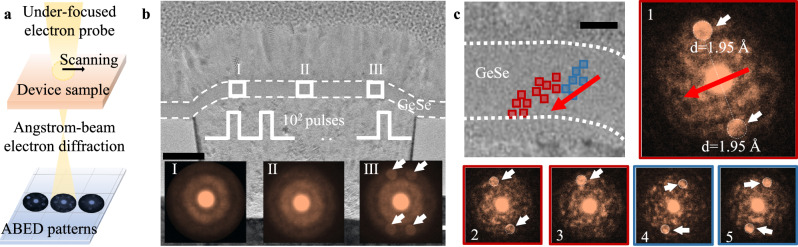
Field-orchestrated CF formation via atomic dipolar chain alignment. **a** Schematic of ABED for spatiotemporal imaging of field-induced ordering motifs. **b** Cross-sectional Cs-TEM image of cycled GeSe device (10^2^ cycles) with ABED diffraction patterns from three regions. Region III exhibits sharp diffraction spots, indicating local atomic ordering. **c** Magnified ABED view of Region III, revealing a CF path formed by aligned amorphous motifs. The red squares (numbered 1–3) and blue squares (numbered 4–5) exhibit two types of diffraction spots (white arrows). Red arrows approximate current flow direction. Scale bars: 50 nm (**b**) and 10 nm (**c**).

Our work starts by answering the central question as to whether threshold switching inherently involves structural evolution. The T-shape device with an amorphous GeSe layer (~20 nm) in a TiN/GeSe/TiN structure, as shown in Supplementary Fig. 1, was switched 100 times. Subsequently, the device was promptly and meticulously sectioned into a Cs-TEM slice (see “Methods” section). The ABED technique, which was initially developed to observe local atomic arrangement in metallic glass^26^, is employed to probe electric-field-driven local atomic ordering in amorphous GeSe. Figure 1a shows the schematic of ABED acquisition based on the 4D-STEM technique for spatiotemporal imaging of field-induced motifs on the GeSe active layer.

Scanning across the entire region between the TiN electrodes (demarcated by white lines in Fig. 1b), we integrate diffraction patterns from three zones. Pristine regions exhibit broad diffraction rings, confirming intrinsic amorphous disorder. Strikingly, post-switching region III reveals sharp diffraction spots, with *d*-spacing measured as 1.95 ± 0.05 Å (Fig. 1b, bottom), a signature of field-induced local atomic ordering. Region III is a residual ordered domain originating from field-induced ordering during prior ON-states. High-resolution ABED tracking (Fig. 1c) further identifies two distinct motif configurations: (i) linear chains (66% as red squares) with diffraction vectors perpendicular to the current flow (*θ* = 90 ± 5°), forming straight filament segments; (ii) kinked branches (34% as blue squares) showing ±15° diffraction vector shifts, indicative of field-gradient-driven reorientation. The CF formation was predicted at *V*_th_ by electrical characterization^27^. These observations reveal a hierarchical CF growth mechanism: electric-field-promoted dipolar alignment first organizes Ge–Se dipoles into linear chains, after which CFs nucleate and propagate perpendicular to the chain axis, with stochastic branching emerging that results from a slightly different orientation of dipolar chains. The measured 1.95 Å spacing, confirmed by the EF-MD simulations to correspond to Ge–Se bond chain configurations under electric field (vide infra), suggests non-equilibrium chain stabilization via dipole-dipole interactions. The branched current path (Fig. 1c, blue squares) stems from field-driven dipolar alignment navigating through amorphous heterogeneities, where transient angstrom-scale local ordered motifs bridge ultrafast dipolar switching and macroscopic threshold dynamics.

### Electrical validation of filament-governed switching

**Fig. 2:**
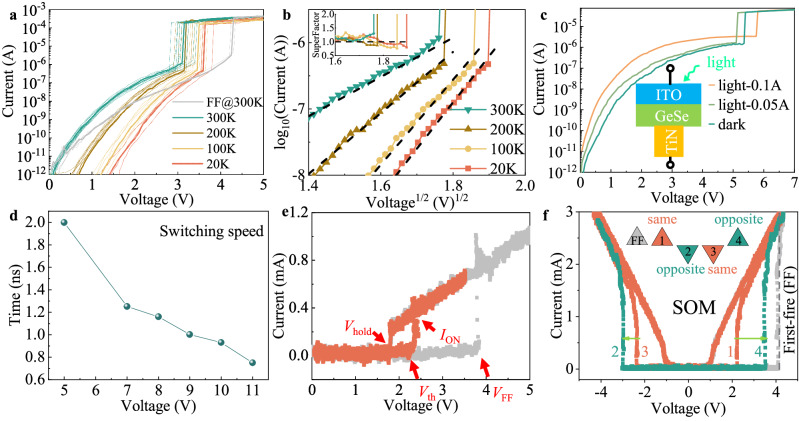
Electrical validation of CF-governed threshold switching. **a** Temperature-dependent current–voltage characteristics (20–300 K) showing PF emission behavior (log_10_ *I*∝*V*^1/2^) in the subthreshold regime. **b** Linear log_10_ *I*–*V*^1/2^ relationships across temperatures (20–300 K), with inset quantifying the dimensionless enhancement factor (actual/PF-predicted current ratio). The dashed lines denote the predicted PF values. **c** Photoresponse under 516.8- and 637-nm illumination: laser-induced photocurrent generation with threshold voltage invariance. **d** Switching time (*t*_on_) versus voltage for GeSe-based OTS devices, demonstrating power-law acceleration (*t*_on_ ∝*V*^−2.56^) adapted from ref. ^28^. **e** Voltage activation sequence: initial forming at *V*_FF_, threshold switching at *V*_th_, and sustained operation at holding voltage *V*_hold_. **f** Polarity-dependent threshold hysteresis under alternating-polarity pulses: matching-polarity sequences (pulses 1, 3) reduce thresholds (2.2 V, −2.3 V), while polarity reversal (pulses 2, 4) restores higher thresholds (−3.0 V, 3.5 V).

This section establishes structural reorganization as the governing mechanism for CF-mediated threshold switching through electrical characterization of three diagnostic signatures: (i) field-driven defect generation in the subthreshold regime, (ii) threshold insensitivity to carrier population modulation, and (iii) structural-state-dependent switching dynamics. In the subthreshold regime, electric field-induced atomic rearrangement generates midgap defect states^28^, a conclusion supported by the Poole–Frenkel (PF) theory with a different exponential current-voltage relationship (Fig. 2a) for the pre- and post-forming conditions. Direct visualization of field-induced amorphous network reconfiguration via the ABED diffraction (Fig. 1) results and atomistic simulations in refs. ^20,29^ confirm defect creation through structural distortion. The temperature-dependent threshold voltage reduction (3.6 V at 20 K to 3.1 V at 300 K) correlates directly with enhanced thermal mobility of the atoms, enabling thermally-assisted CF formation. Notably, to remove the first-fire curves, triangular pulses are implemented prior to the DC *I*–*V* characterization.

Analysis of photoresponse experiments on the systems reiterates the primary structural, not electronic, contribution to the threshold switching. Compared to the dark condition, illumination with 516.8-nm and 637-nm lasers generated photocurrent (Fig. 2c). Although the photoresponse at 637 nm is slightly weaker than that at 516.8 nm, both wavelengths produced a significant increase in photocurrent relative to the dark background. The threshold voltages under illumination remained largely unchanged, a behavior consistent with that observed in the Te_40_As_35_Ge_7_Si_18_ system^11^. This invariance, combined with little superexponential current enhancement, illustrating no carrier multiplication phenomenon, near switching (dimensionless factor defined as actual/PF-predicted current ratio = 1.30 at 300 K versus ≈1 at cryogenic temperatures, Fig. 2b), demonstrates that neither electronic avalanche nor photon-driven carrier injection can govern the switching. It is noted that, compared to ref. ^28^, the absence of avalanche multiplication herein is attributed to both the current-limiting series resistor (Rs = 10 kΩ) and active thermal stabilization (<±0.01 °C fluctuation) implemented to suppress current heating near the switching. Instead, these results highlight that atomic bond reconfiguration (requiring energy surpassing electronic excitation) gates the switching.

Definitive structural signatures appear in dynamic switching behavior. The voltage-accelerated kinetics (*t*_on_ ∝ *V*^−2.56^, *R*^2^ > 0.99, adapted from ref. ^28^) exhibit a switching time reduction from 2.0 ns at 5 V to 0.75 ns at 11 V (Fig. 2d). This voltage dependence reflects electric-field-induced lowering of energy barriers for atomic rearrangement, demonstrating a non-Arrhenius mechanism where the electric field dominates over thermal energy barriers. This field-driven mechanism governs also the voltage-dependent suppression of switching delay^11,30^, where elevated bias destabilizes metastable configurations while preserving constant OFF-current prior to switching.

In Fig. 2f, five triangular pulses are performed to study *V*_th_ asymmetry. The |*V*_th_| values for the first (1) and third (3) pulses are smaller than for the second (2) and fourth (4). The first two have the same polarity, while the last two have opposite polarity. Stabilization of reconfigured dipolar order through holding voltage (*V*_hold_, Fig. 2e) and polarity-dependent threshold asymmetry (2.2–3.5 V hysteresis in alternating-polarity pulses, Fig. 2f) originate from polarity-controlled polarization reversal of the dipole configurations. The *V*_th_ drift, measured with the coefficient of 53.4 mV/dec, stems from polarization relaxation dynamics, where the dipolar order is gradually lost upon voltage removal.

### Dipolar alignment drives atomic ordering

**Fig. 3:**
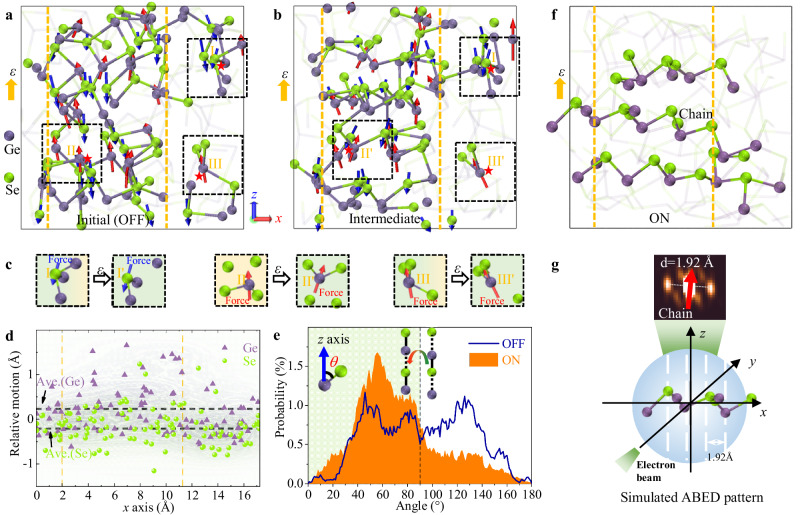
Field-driven dipolar alignment governs local atomic ordering. **a** Initial disordered bonding network (0 ps) with random Ge (blue)–Se (green) orientations (bond cutoff: 2.8 Å). **b** Intermediate state (1.2 ps) under 0.04 V/Å electric field: Selective bond break (Se ↓ –Ge↑ bonds around I–III) and reformation (Ge ↓ –Se↑ bonds around I’–III’) establish field-aligned Ge ↓ –Se↑ bonds. Red (upward) and blue (downward) arrows in (**a**, **b**) denote oppositely directed electric field forces acting on Ge and Se atoms, respectively. **c** Bond breaking and forming during the I → I’, II → II’, and III → III’ transitions. **d** Atomic displacement asymmetry: Ge upward shift (+0.23 Å) vs. Se downward shift ( − 0.21 Å), confirming dipolar polarization. **e** The angular distribution of Ge–Se bonds relative to the E-field (*z*-axis): initial-state (0 ps, blue, symmetric distribution), isotropic transitions to ON-state (orange, the least at 90°), alignment along *z*-axis. **f** EF-MD-simulated field-aligned Ge ↓ –Se↑ chains. **g** Multislice simulated ABED patterns of the chain match the experimental observation of linear chains being perpendicular to the current flow (red arrows).

In this section, we perform EF-MD simulations on a 200-atom amorphous GeSe model (using *NVE* ensemble with initial temperature at 300 K) to probe field-induced dipolar alignment and local atomic ordering, employing finite periodic electric fields and Berry-phase polarization theory within the CP2K framework^31^. Under a 0.04 V/Å E-field, the Ge and Se atoms exhibit anti-parallel displacements relative to the field direction (*z*-axis), with average shifts of +0.23 Å (Ge) and −0.21 Å (Se) (Fig. 3d). The calculated Ge/Se displacements match experimental data from device failure conditions (Supplementary Fig. 2), validating the simulated bond reconfiguration dynamics. This motion breaks unfavorable Ge(up)–Se(bottom) bonds (Fig. 3a) while forming aligned Ge(bottom)–Se(up) bonds (Fig. 3b), directly increasing the dipole moment (vide infra). Atoms I–III exhibit sequential bond inversion: Se(bottom)–Ge(up) bonds break while Ge(bottom)–Se(up) bonds are formed, demonstrating field-driven, dynamical directional rebinding, as shown in Fig. 3c. This picosecond bond reorientation dynamics reveal a non-Arrhenius mechanism, fundamentally distinct from thermally activated processes governed by Arrhenius kinetics. Notably, the regions exhibiting maximal bond reorientation, identified by tracking Ge/Se atoms with largest displacements (electric force vectors marked by arrows in Fig. 3a, b) and Ge(up)–Se(bottom) bond inversion, spatially overlap within the CF locations (vide infra). This result establishes direct correlation between the atomic-scale bond dynamics and the device-scale switching pathways.

While pristine Ge–Se systems exhibit disordered Peierls-like bond-length disproportionation (long/short pairs corroborated by the angular-limited bond correlation shown in Supplementary Fig. 3)^32^, the electric field selectively elongates unfavorable Ge(up)–Se(bottom) short bonds, prompting their breaking and rebinding as Ge(bottom)–Se(up), resulting in directional alignment. Angular analysis of Ge(initial)-Se(terminal) bonds relative to the *z*-axis (*θ* in Fig. 3e) reveals polarization-induced alignment: the initial symmetric distribution (*θ* around 90°, blue) shifts to a *θ* < 90° dominance (orange), demonstrating field-driven bond reorientation. This directional preference templates the subsequent 1D chain assembly pathway (Fig. 3f). Figure 3g shows that the simulated ABED pattern for the *xz*-plane chain exhibits two sharp diffraction spots (1.92 ± 0.05 Å spacing) orthogonal to the current direction (*z*-axis), quantitatively matching experimental ABED data (Fig. 1b, c). This geometric correlation confirms that the Ge–Se chains oriented perpendicular to the electric field (current flow direction) mediate structural ordering, while individual Ge–Se bonds tend to align parallel to the field. This demonstrates a dual alignment mechanism enabling both dipolar polarization and CF formation.

Theoretical simulations indicate that Ge–Ge chains are obtained by fast quenching in Ge-based amorphous chalcogenides^33,34^. However, their structural characteristics conflict with experimental observations: the Ge–Ge bond length (~2.8 Å) and Ge–Ge–Ge bond angles (typically >109°28’, deviating from ideal *sp*^3^ hybridization) yield calculated ABED *d*-spacings exceeding 2.27 Å (via *d* = 2.8 × sin(109.5°/2)), as demonstrated by the three Ge–Ge–Ge chains in Supplementary Fig. 4, all exhibiting d-spacings »1.95 Å. In contrast, Ge–Se bond chains exhibit higher thermodynamic stability (bond energy: 51.5 vs. 37.6 kcal/mol for Ge–Ge)^35^ and structural compatibility with experimental data. As shown in Fig. 3f, Ge–Se chains feature a bond length of 2.54 Å and a Ge–Se–Ge angle of 98°, slightly distorted from the 90° *p*–*p* orbital interaction, resulting in a calculated *d*-spacing of 1.92 Å (*d* = 2.54 × sin(98°/2)), as shown in Fig. 3g, closely matching the experimental value of 1.95 Å. These results collectively exclude Ge–Ge chains as contributors to the observed ABED signal and confirm Ge–Se chains as the dominant structural motifs.

### Threshold switching and asymmetric switching voltage

Subsequently, we investigate how electric-field-driven amorphous structural reconfiguration mediates CF formation and demonstrate that polarity-dependent dipolar orientation governs the *V*_th_ asymmetry. A SOM crossbar array comprises OTS layers and electrodes^10^, as shown in Supplementary Fig. 2e. Application of a voltage pulse between the selected word line (WL) and bit line (BL) triggers CF formation^36,37^. To unravel the atomic-scale mechanisms of both OFF-to-ON transitions and polarity-induced *V*_th_ asymmetry, we perform extensive EF-MD simulations. Our primary system is a 200-atom amorphous GeSe model, supplemented by other models in Supplementary Fig. 5, notably a larger 3600-atom one.

**Fig. 4:**
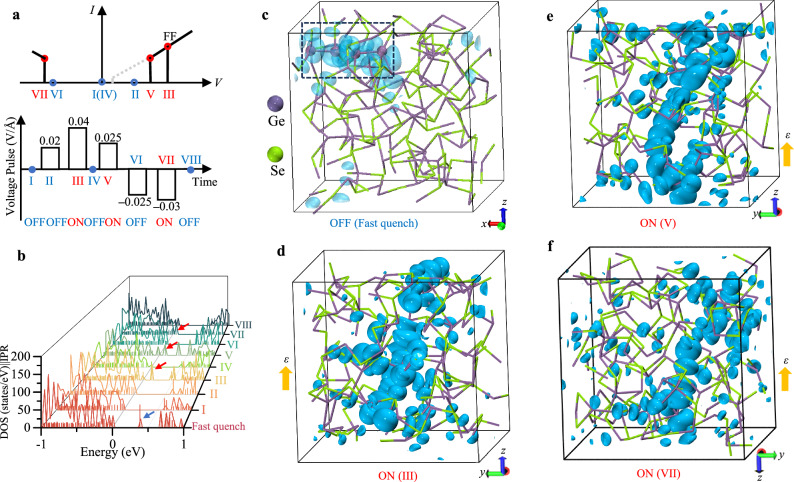
Field-stabilized dipolar order mediates threshold switching and *V*_th_ asymmetry. **a** To obtain the structural features during the turn-on process, EF-MD simulations are performed at different voltage conditions along the *I*–*V* curve by applying voltage pulses. Schematic of the electrical operation sequence: (I) initial OFF → (II) sub-threshold → (III) 0.04 V/Å (ON, FF) → (IV) 0 V/Å (OFF) → (V) 0.025 V/Å (ON, same polarity) → (VI) − 0.025 V/Å (OFF, opposite polarity) → (VII) − 0.03 V/Å (ON, opposite polarity). **b** DOS for eight metastable configurations (labeled I–VIII) post-pulse operation, compared to a fast-quenched reference. All DOSs represent Gaussian-broadened (*σ* = 0.01 eV) projections of discrete molecular orbitals. IPR (×2000) highlights localization-to-delocalization transitions, with IPR vertical lines also marking molecular orbital energy levels. **c**–**f** MO charge density isosurfaces (±0.01e Bohr^−3^): **c** fast-quenched reference with localized defect states (OFF, green arrow in (**b**)); **d**–**f** Field-aligned states (III, V, VII) exhibit delocalized MOs along CFs (ON, red arrows in (**b**)), confirming dipolar-ordering-enabled high conductivity pathways.

To obtain the *V*_FF_, the EF-MD simulations are carried out by changing square-voltage pulses applied along the *z*-axis with the strength changing from 0 V/Å to 0.06 V/Å for 150 ps. We define the turn-on state as a CF is formed, characterized by the approach of the lowest unoccupied molecular orbital (LUMO) toward the highest occupied molecular orbital (HOMO). The field-reduced band gap, i.e., a small (*E*_LUMO_−*E*_HOMO_) value, remains consistent with the Berry phase polarization framework, as systematically validated in Supplementary Figs. 6 and 7 under dense k-point sampling. The simulations show that threshold switching initiates at *V*_FF_  = 0.04 V/Å (onset at 83.31 ps, Fig. 4a), characterized by delocalized MOs near the Fermi level (Fig. 4b). Sustaining the ON-state requires a holding voltage *V*_hold_ =0.005 V/Å. After 4.5 ps MD-relaxation without E-field (Step IV) to an OFF state, the subsequent switching-ON occurs at *V*_th_ = 0.025 V/Å (state V). Strikingly, negative polarity demands a higher threshold |*V*_th_| = 0.03 V/Å (Fig. 4a) (state VII).

The polarity-dependent *V*_th_ shift can be exploited in the SOM application. The asymmetry arises from different dipole-orientation-dependent CF growth: States III and V share the same dipole orientations, and state VII exhibits the opposite dipole orientation. The residual polarization in state V reduces its *V*_th_ relative to state III, while reorienting dipoles to the opposite direction (state VII) requires a higher *V*_th_ to overcome the energy barrier. This aligns with the intuitive explanation of the asymmetry, where directional conduction clusters exist^6^. This polarity contrast is in line with the experimental polarity-induced *V*_th_ shifts (Fig. 2f), although simulated values are ~2× larger, a discrepancy attributable to the 150-ps simulation window in the finite size of the simulation models versus experimental nanosecond-scale switching.

Figure 4c–f reports the spatial distribution of LUMOs highlighted in Fig. 4b, corresponding to CF formation in the ON states (III, V, VII) and a localized defect state in the fast-quenched OFF-state model. Notably, the ON-state MOs exhibit delocalization along the electric field direction (*z*-axis), forming a conductive channel ~1 nm in diameter (Fig. 4d–f). The formation of CF in Fig. 4d is driven by the field-favored Ge–Se chain (Fig. 3f). The dipolar chain primarily serves as a structural template, which induces a localized strain and electrostatic potential field. This field promotes bond distortion and coordination defects (e.g., under-coordinated Ge^3+^) at the chain-matrix interface. These structural perturbations create delocalized electronic states near the Fermi level, thereby guiding the nucleation and subsequent CF growth. The resultant high conductivity is thus an intrinsic property of the matured, percolating CF, rather than of the precursor chain itself.

In contrast, the OFF-state defect MOs remain localized (Fig. 4c), such as in the homopolar Ge–Ge bonds or their bond chains^33^. Supporting this distinction, the significantly lower inverse participation ratio (IPR) of the ON-state molecular orbitals (around 0.01, Fig. 4d) compared to the localized defect state (0.032, Fig. 4c) confirms the delocalized electronic nature of the CF. This is consistent with ref. ^38^, in which IPR values of 0.01–0.02 (0.03–0.06) were reported for (partially) delocalized states; it should be noted, however, that the larger system size in that study results in generally lower IPR ranges. A detailed IPR comparison of these states is provided in Supplementary Fig. 8. The slightly larger IPR values of both the LUMO and HOMO, compared to the deep states, can be attributed to the quasi-one-dimensional (quasi-1D) character of the CF structure, analogous to the electronic states found in quasi-1D or quasi-2D phase-change materials. The ON states (III, V, VII) exhibit no obvious localized states around the limited number of Ge–Ge bonds, as evidenced by the slower quenching rate and a minor atomic displacement of ~0.2 Å during the switching process.

For the III (ON) state, the HOMO (Supplementary Fig. 9) is delocalized within the same conductive channel as the LUMO depicted in Fig. 4d. The same delocalized region enables facilitated generation of hole-electron carriers via electronic excitation from the HOMO to the LUMO, thereby inducing high conductivity along the channel. Further validation via hybrid-DFT HSE calculations (Supplementary Fig. 10) corroborates the delocalization of the LUMO wavefunction within the CF. Notably, the CF orientation aligns with the applied electric field direction (*x*-axis in Supplementary Fig. 11), mirroring the polarity-dependent dipolar alignment mechanism found in Fig. 3. Large-scale atomistic simulations (5.2 × 5.2 × 3.5 nm^3^, 3600 atoms) in Supplementary Fig. 5 uncover ultrafast diameter expansion of CFs that defies classical nucleation timescales, with direct experimental evidence of pancake-shaped filaments (aspect ratio < 1, ref. ^27^) revealing geometric constraints. Identical switching signatures of the 3.5 nm-thick devices, as shown in Supplementary Fig. 12, prove our simulated 5.2 × 5.2 × 3.5 nm^3^ process can reflect realistic device-scale physics. Altogether, these results establish that field-driven dipolar reconfiguration enables directional growth of CFs, whose high electronic conductivity stems from both reduced gap and delocalized LUMOs in stark contrast to the large gap, localized defect states of the OFF-state.

The polarity-dependent *V*_th_ asymmetry, governed by dipole-aligned CF kinetics (Figs. 3 and 4c–f), enables robust binary memory operation in SOM crossbars. Distinct SET (+*V*_*th*_) and RESET (−|*V*_*th*_|) transitions (Fig. 2f) permit deterministic 0/1 state switching, while field-directed CF growth (Fig. 4d–f, Supplementary Fig. 5) ensures reliable non-volatile bit storage. By directly linking atomic-scale dipolar reconfiguration to macroscopic selector functionality (Fig. 2e, f), this work establishes a universal mechanism for designing amorphous chalcogenide-based memory with engineered threshold voltage asymmetry. The revealed structure-property relationship enables rational screening of SOM systems exhibiting field-free dipolar metastability by quantitatively evaluating zero-bias dipole relaxation dynamics.

### Dipolar response and polarization dynamics

The ultrafast, non-Arrhenius kinetics of field-induced dipolar ordering in amorphous GeSe, evidenced by bond chain configurations in Fig. 1c and Fig. 3f, deviate from classical nucleation-mediated crystallization. In contrast to the latter, this process is driven by coordinated dipolar reconfiguration, where electric fields induce polarity-dependent bond realignment. To probe the existence and dynamic evolution of dipoles in this mechanism, we integrate EF-MD simulations with dielectric spectroscopy, revealing how dipolar interactions govern both the energy landscape under electric field and the material’s macroscopic polarization response.

**Fig. 5:**
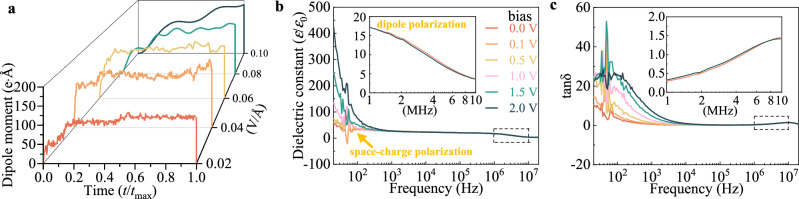
Dipolar response and polarization dynamics in amorphous GeSe. **a** Field-dependent evolution of net dipole moment (normalized time *t*/*t*_max_) from EF-MD simulations. *t*_max_ was set to 1000 ps for the OFF-state field strength (0.02 V/Å), while *t*_max_ (=*t*_on_) decreased exponentially with increasing field: 83.32 ps at 0.04 V/Å, 9.45 ps at 0.06 V/Å, 1.29 ps at 0.08 V/Å, and 0.489 ps at 0.10 V/Å. This demonstrates field-accelerated dipole reorientation kinetics, where higher electric fields dramatically reduce the timescales of structural reconfiguration. **b**, **c** Frequency-dependent dielectric constant (**b**) and loss tangent (**c**) under DC biases (0–2 V). Low-frequency (<10^3 ^Hz) dielectric enhancement at higher biases (insets) confirms field-induced dipolar polarization, while high-frequency suppression reflects kinetic limitations in dipole reorientation.

Under varying electric fields (0.02–0.10 V/Å), EF-MD simulations reveal that threshold switching accelerates with increasing field strength. At 0.04 V/Å, switching initiates at 83.31 ps, while at 0.08 V/Å, it reduces to 1.29 ps, a timescale comparable to THz-driven processes^16^ and intrinsic atomic vibrations (~3.14 ps, Supplementary Fig. 13). This field-dependent switching time reflects the non-Arrhenius nature of threshold switching. This is possible because the electric field, through alignment of the dipole moment (*μ*), changes the energy barriers governing the nuclear dynamics. The value of *μ* is calculated via:

1 $$\mu=4\sum {r}_{{{\mathrm{Ge}}}}+6\sum {r}_{{{\mathrm{Se}}}}-2{\sum }_{i}{W}_{i}$$

(where *r*_Ge_, *r*_Se_, and *W*_*i*_ denote positions of Ge/Se atoms and Wannier centers), increases sharply at early stages (low-barrier Ge‒Se bond exchange) and gradually thereafter (high-barrier rearrangements). Figure 5a reveals that the dipole moment exhibits rapid initial alignment through low-energy-barrier dipolar reconfiguration, followed by a gradual increase due to slower structural relaxation. This behavior aligns with the reduction in free energy (*E*_*F*_ = *E*_*P*_ + *W*_*E*_), where the electric field-reduced potential energy (*W*_*E*_ = ‒Δ*μ·ε,* with Δ*μ* denoting the change of dipole moment and *ε* the applied electric field) counteracts the rising structural potential energy (*E*_*P*_, Supplementary Fig. 14). The increased *E*_P_ arises from a fundamental interplay: CFs preferentially nucleate in regions of higher dipolar order (Figs. 3b and 4e), yet this local order conflicts with the surrounding disordered matrix, inducing bond distortion and coordination defects (e.g., undercoordinated Ge^3+^), as shown in Supplementary Fig. 15. This structural frustration generates delocalized electronic states near the Fermi level, enabling high conductivity through CFs percolating across the material. In other ionized molecules, this energy compensation mechanism also mirrors field-stabilized conformational states, where external fields modulate energy landscapes to favor specific configurations^39^. Temperature rises from initial 300 K to 401 K at 0.04 V/Å, which further confirms energy dissipation during the reconfiguration. In a separate EF-MD simulation starting at 50 K, a CF formed, accompanied by a temperature rise to 274 K, as shown in Supplementary Fig. 16.

To experimentally confirm dipoles, we measure the frequency-dependent dielectric constant of 162-nm-thick amorphous GeSe using a circular capacitor (TiN electrodes, area: 0.21 mm^2^). Two polarization regimes emerge: (1) Dipole polarization (1 kHz–10 MHz): The dielectric constant decreases from 17.1 to 3.7 as frequency increases (Fig. 5b), while dielectric loss (tan *δ*) rises from 0.31 to 1.45 (Fig. 5c). Above 1 MHz, the dipoles lag behind the alternating field, reducing the polarization efficiency. The slower dipole relaxation time measured experimentally, compared to the picosecond switching time observed in EF-MD simulations, results from the very weak electric field applied, which confines the measurement to the linear response regime and thus probes the average behavior of the entire dipole population. (2) Space-charge polarization (20–500 Hz): Extreme dielectric loss (tan*δ* ≫ 1) indicates leakage currents from charged atom migration (Fig. 5c). The bias-modulated frequency responses, governed by Poole–Frenkel^40^, reveal a field-reduced migration barrier of charged atoms. This is in line with the progressive dipole-ordering dynamics captured in EF-MD simulations during threshold activation (Fig. 5a).

The interplay between dipole and space-charge polarization determines the threshold switching dynamics across different timescales. At low field, low-energy-barrier dipole polarization (local Ge‒Se bond realignment) occurs rapidly, while space-charge polarization (long-range atom migration) progresses slowly. However, under high fields near *V*_th_, field-driven atomic migration (Supplementary Fig. 2) facilitates long-range dipole enhancement, stabilizing the field-aligned ordered state (Figs. 1c and 3f). Crucially, the slow, field-accelerated migration of charged atoms, evidenced by the gradual increase in dipole moment at later stages (Fig. 5a), acts as the rate-limiting step for threshold switching. This dual-polarization mechanism not only resolves *V*_th_ drift during operation but also defines a materials design strategy: stabilizing *V*_*th*_ by suppressing structural degradation via short-pulse-controlled charged atom migration in tailored aging protocols^41^.

In summary, our discovery of field-programmable configuration dynamics as a universal driver of atomic-scale structural ordering redefines threshold switching in chalcogenides, establishing a materials design paradigm that bridges quantum phase control and advanced electronic functionality. The non-Arrhenius kinetics of field-driven dipolar alignment, exemplified by picosecond Ge‒Se bond reorientation, suggest transformative potential in stabilizing exotic quantum states. For instance, pressure-induced superconductivity in amorphous Sb_2_Se_3_^42^, where bond distortion enables electronic delocalization, mirrors how field-controlled structural reconfiguration could nucleate metastable superconducting states in disordered systems. Simultaneously, field-induced covalent bond distortion enables precise modulation of polar order in amorphous matrices, aligning with predictions for ferroelectric glass state^43^, thereby offering a platform to realize field-engineered quantum phases. By unifying correlated electron physics, structural adaptability, and neuromorphic principles, this work provides a blueprint for quantum materials where electronic and structural degrees of freedom cooperatively evolve. Future studies employing femtosecond-field pulses (structural precursors) and attosecond probes (electronic delocalization) could unravel the hierarchy of structure-driven electronic transitions, akin to resolving lattice-mediated Cooper pairing in strain-tuned superconductors^44^.

## Methods

### Device preparation

T-shaped cells with 200-nm pillar TiN bottom electrodes were fabricated by 45 nm CMOS technology. The cells were etched for 20 min with Ar ions to clean the surface oxide layer. Then 20-nm GeSe amorphous chalcogenide and 40-nm TiN top electrodes were sequentially deposited by physical vapor deposition, with sputtering rates of 2 nm/min at 20 W and 0.5 nm/min at 50 W, respectively. The amorphous GeSe film was deposited on a 40-nm TiN bottom electrode. To deposit GeSe and TiN films, we use an alloy target to sputter. Before the deposition, the pressure is 3 × 10^−4 ^Pa. During the deposition, the introduced Ar for sputtering is at a flow rate of 50 SCCM (cubic centimeters per minute at standard temperature and pressure) with a 2 × 10^−1 ^Pa background pressure. The substrate is at room temperature. After the sputtering, the GeSe film is in the amorphous state, and the top TiN electrode is in the crystalline phase. For our magnetron sputtering equipment, the target-to-substrate distance is 150 mm.

### Angstrom-beam electron diffraction details

Samples for spherical aberration-corrected transmission electron microscope (Cs-TEM, JEOL GRAND ARM 300 F) studies were fabricated using a focused ion beam (FIB, Helios G4), including low-energy cleaning to remove possible damage. To achieve an electron beam with angstrom-level parallelism, a 10 µm condenser lens aperture was employed. The convergence angle of the electron beam was adjusted through optimizing the beam path, resulting in a value of 1.83 mrad and achieving a beam size of 6.56 Å.

### Electrical measurement

At room temperature, the device performance was characterized by a Keithley 4200A-SCS instrument. The device performance was characterized by applying pulses through a Keithley 4200A-SCS instrument rather than DC testing because of the significant damage to the device caused by DC testing. Supplementary Fig. 17 shows the circuit diagram for device testing.

Electrical characterization at cryogenic temperatures was conducted using a Lake Shore Model 336 cryogenic probe station equipped with a closed-cycle helium refrigerator (base temperature: 4.2 K). The vacuum environment was maintained below 1 × 10^−3 ^Pa via a combination of turbomolecular and ion pumps to minimize thermal drift and gas adsorption. Samples were mounted on a copper cold finger using indium cold-welding, ensuring thermal contact resistance <1 mΩ. Temperature stability was controlled within ±0.05 K across the 20–300 K range using a Lake Shore 336 controller with PID-optimized feedback. DC current–voltage (*I*–*V*) measurements were performed with a Keithley 4200A-SCS Parameter Analyzer (SMU modules configured in 2-wire mode). Voltage sweeps were applied at a rate of 0.01 V s^−^¹, and current compliance was set to 1 mA to prevent device breakdown. All data were acquired under thermal equilibrium conditions (temperature fluctuation <0.1 K over 5 min). For laser illumination experiments, a single-mode fiber-pigtailed laser (Thorlabs Inc.) was integrated with devices featuring 20 nm indium tin oxide (ITO) top electrodes, which were deposited via magnetron sputtering, replacing the 40 nm TiN electrodes to enable optical transparency. Prior to testing, devices underwent 140 °C annealing in a N_2_ atmosphere for 15 min to reduce ITO sheet resistance, which consequently reduced *V*_th_ values due to a modified field distribution across the active layer.

The relaxation polarizations, including space-charge polarization and dipole polarization, were measured by the Keysight E4990A Impedance Analyzer from 20 Hz to 120 MHz. During the measurement, bias voltages of 0 V, 100 mV, 500 mV, 1 V, 1.5 V and 2 V were applied to activate the trapped charged particles, which affects the space-charge polarization below 1 kHz. From 1 kHz to 10 MHz, dipole polarization is the main relaxation polarization.

### Computational details

Ab initio molecular dynamics (AIMD) simulations were carried out using the Quickstep module of the CP2K package^45^. Goedecker–Teter–Hutter norm-conserving pseudopotentials and a gaussian-plane waves basis set were used, with a kinetic energy cutoff of 320 Ry for the plane waves. Throughout the simulations, the orbital transformation (OT) algorithm, as implemented in CP2K/QS, was used to achieve SCF convergence by optimizing the wavefunction via orbital rotations. This method requires integer occupations and a finite band gap to proceed effectively. Based on a dual basis of atom centered Gaussian orbitals and plane waves, the Gaussian and plane waves (GPW) method expands the Kohn–Sham orbitals using double-ζ valence plus polarization (DZVP) Gaussian basis sets, and the electronic density using plane waves. The Perdew−Burke−Ernzerhof (PBE) functional^46,47^ with DFT-D3 corrections^46,47^ was used. In all the simulations, only the gamma point was sampled. The valence electrons are *4s*^*2*^*4p*^*2*^ for Ge and *4s*^*2*^*4p*^*4*^ for Se. To obtain the zero-pressure-amorphous model, molecular dynamics (MD) simulations in the *NPT* ensemble were carried out using the melt-quenching method with a 200-atom system. The system was first melted at 2000 K for 100 ps and then quenched to 300 K over 150 ps. The finally obtained cubic box has a length of 17.24 Å. At 1000 K, the pair distribution function shown in Supplementary Fig. 18 is consistent with experiment^48^. For the simulations with an applied electric field, the *NVE* ensemble was used with initial temperature at 300 K. The relaxation processes were simulated using the *NVT* ensemble with the Nose-Hoover thermostat. In all the MD simulations, the time step was 3-fs.

Starting from a unit cell of 1.7 nm × 1.7 nm × 1.7 nm (200 atoms), we studied three supercells: 3.5 nm × 3.5 nm × 1.7 nm (800 atoms), 3.5 nm × 3.5 nm × 5.2 nm (2400 atoms), and 5.2 nm × 5.2 nm × 3.5 nm (3600 atoms). We relaxed the supercells for 3 ps at 500 K, 3 ps at 400 K, and 6 ps at 300 K. To shorten the filament-forming time, an electric field of 0.06 V/Å was used for the 3.5 nm × 3.5 nm × 5.2 nm and 5.2 nm × 5.2 nm × 3.5 nm models.

### Electric field forces

Electric field forces are the key to understanding the reconfiguration process, as at high fields, these forces drive systems to a new energy landscape. This mechanism is analogous to advanced sampling techniques that utilize biased forces (here, the electric force *q****ε***), such as umbrella sampling^49^ and metadynamics^50^ which modify the energy landscape to enhance the sampling of rare events, including crystallization and chemical reactions. However, under high electric fields, electronic self-consistency process becomes difficult due to the formation of a large delocalized region.

To avoid the difficult convergence during AIMD simulations under high electric field strength, we take two steps: first, the Born effective charge (*Z*^∗^) of individual atoms under a low electric field (*E* = 0.5 MV/cm) was computed, with methodological validation detailed in Supplementary Fig. 19; second, we wrote a separate code implementing the Velocity–Verlet algorithm to update the position and velocity of each atom, accounting for the additional electric-field force due to a higher electric field. In this process, we use the CP2K package to calculate forces both with and without an electric field. Then, we update the position and velocity according to the Velocity–Verlet algorithm externally to CP2K with new code.

The first half of the Velocity-Verlet algorithm updates atomic velocities by half step using the new forces:

2 $$v\left(t+\frac{1}{2}\Delta t\right)=v\left(t\right)+\frac{F(t)}{2m}\Delta t$$

3 $$F(t)=F_0(t)+F_E(t)$$

where *F*_0_ and *F*_E_ are the force without and with the electric field, respectively; *m* is the mass of each atom. Here, we at first obtain the electric-field force *F*_E_(0.005) at 0.005 V/Å; then, the extrapolated electric-field force is obtained as (*E*_Field_/0.005) × *F*_E_(0.005). Figure S18 shows (i) the similar Born effective charges obtained under 0.005 V/Å and 0.04 V/Å electric fields, and (ii) the linear change of the forces as the field changes from 0.0 V/Å to 0.04 V/Å, which justifies the use of this strategy.

The second half of the Velocity-Verlet algorithm updates positions after the full step and velocities at half step:

4 $${{{\boldsymbol{x}}}}\left(t+\Delta t\right)={{{\boldsymbol{x}}}}\left(t\right)+{{{\boldsymbol{v}}}}\left(t+\frac{1}{2}\Delta t\right)\Delta t$$

5 $${{{\boldsymbol{v}}}}\left(t+\Delta t\right)={{{\boldsymbol{v}}}}\left(t+\frac{1}{2}\Delta t\right)+\frac{{{{\boldsymbol{F}}}}({{{\rm{t}}}}+\Delta t)}{2m}\Delta t$$

### Electric potential energy

While the electric field is a key condition to achieve and overcome transition state barriers, it also has a significant effect on the stability of matter. When an atom in a compound with different electronegativity acquires a charge through the gain or loss of *q* electrons, electric potential energy (or electric work), *W*_E_, is performed by the product of electric-field force, *q****ε***, and its displacement, ***S***, under the electric field (***ε***). A simple formula can be used to estimate *W*_E_^51^,

6 $${W}_{E}=-q\cdot{{{{\boldsymbol{S}}}}} \cdot {{\upepsilon}}$$

7 $$=-\Delta \mu \cdot {{\upepsilon}}$$

where *q* and Δ*μ* are the charge of atoms and the change of dipole moment, respectively. *W*_E_ results in the change of free energy landscape (*E*_F_) with electric field: at high fields, the electric field-reduced *W*_E_ dominates the total free energy (*E*_F_ = *E*_P_ + *W*_E_), leading to a landscape-tilt-induced barrier collapse that allows for picosecond bond reconfiguration, as shown in supplementary Fig. 14. The electric force, derived from Berry-phase polarization via OT, serves as the driving force.

## Supplementary information

Supplementary Information

Transparent Peer Review file

## Data Availability

All data needed to evaluate the conclusions in the paper are present in the paper and/or the Supplementary Materials. The source data underlying Figs. 1–5 and all Supplementary Figs. (except for supplementary Figs. 4, 17) are provided as a Source Data file (10.6084/m9.figshare.32083272). Additional data related to this paper can be requested from the corresponding authors.
