# Supplementary material for: Non-Arrhenius threshold switching by field-driven dipolar ordering
Source: Nat Commun. 2026 May 13;17:6404. doi: 10.1038/s41467-026-72970-z (PMC13376810; doi:10.1038/s41467-026-72970-z)
Supplement: Supplementary file 1 — Supplementary Information [file 41467_2026_72970_MOESM1_ESM.pdf]

Non-Arrhenius threshold switching by field-driven dipolar ordering

Wen-Xiong Song[1, †], Guangjie Shi[2,†], Qi Hu[3,†], Fan Zhu[4], Tianjiao Xin[2], Ying Chen[5], Sergiu Clima[6], Gilberto Teobaldi[7], Yuhao Wang[1], Wenjian Huang[1], Sannian Song[1,*], Cheol Seong Hwang[8], Li-Min Liu[3], Yan Cheng[2,*] & Zhitang Song[1,*]

[1]State Key Laboratory of Materials for Integrated Circuits, Shanghai Institute of Microsystem and Information Technology, Chinese Academy of Sciences, Shanghai 200050, China;

[2]Key Laboratory of Polar Materials and Devices (MOE), School of Information and Electronic Engineering & School of Integrated Circuits Science and Engineering, East China Normal University, Shanghai 200241, China;

[3]School of Physics, Beihang University, Beijing 100191, China;

[4]Department of Materials Science, Fudan University, Shanghai 200438, China;

[5]Key Laboratory of Inorganic Functional Materials and Devices, Shanghai Institute of Ceramics, Chinese Academy of Sciences, Shanghai 200050, China;

[6]imec, Kapeldreef 75, B-3001 Leuven, Belgium;

[7]Scientific Computing Department, STFC UKRI, Rutherford Appleton Laboratory, Didcot OX11 0QX, U.K.

[8]Department of Materials Science and Engineering and Inter-University Semiconductor Research Center, Seoul National University, Seoul 08826, South Korea.

†These authors contributed equally: Wen-Xiong Song, Guangjie Shi, Qi Hu.

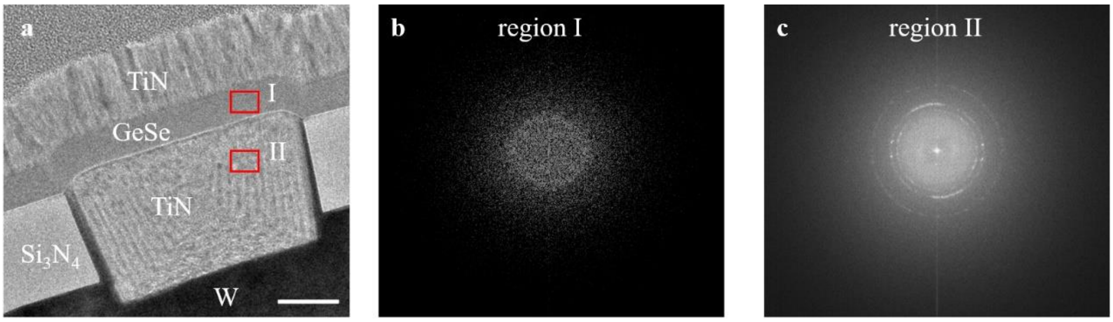

Supplementary Figure 1 Cross-sectional TEM analysis of a 20-nm-thick GeSe device. (a) TEM image of the T-shaped device structure. (b) Fast Fourier transform (FFT) pattern of Region I, confirming the amorphous nature of the GeSe layer. (c) FFT pattern of Region II, corresponding to the crystalline TiN electrode. The scale bar is 50 nm.

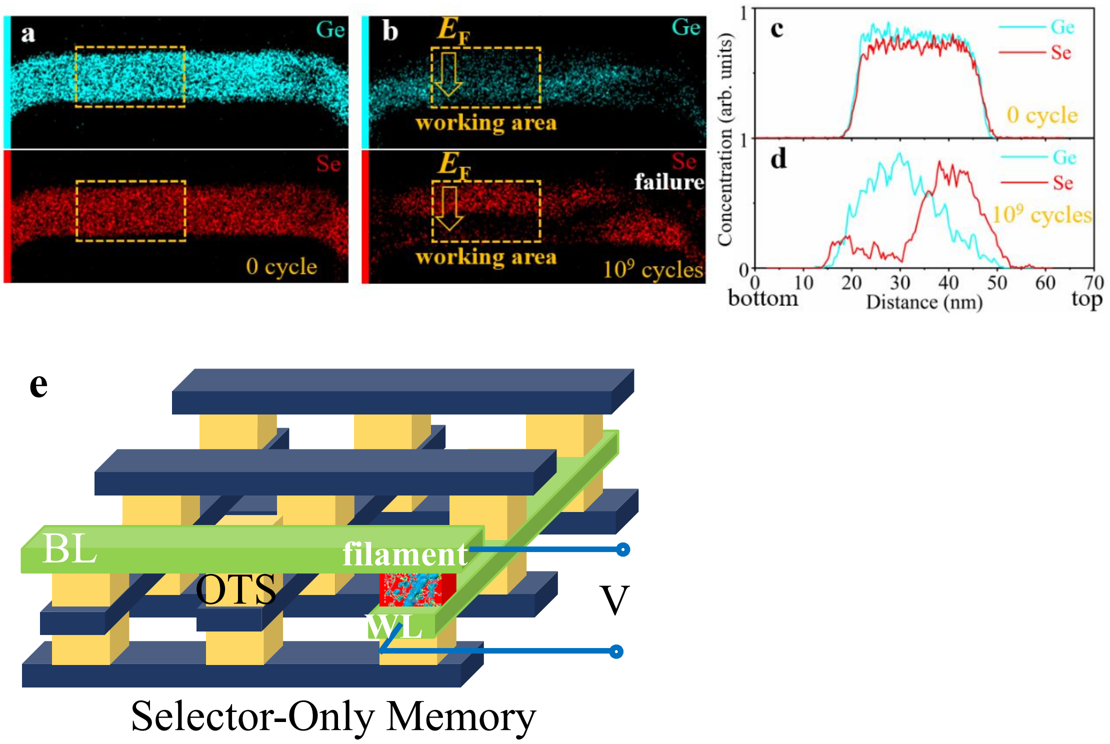

Supplementary Figure 2 The cumulative relative atomic motion during operation in the selector application. (**a**, **b**) EDS elemental mappings for the 0 cycle (**a**) and 10^9 cycles (**b**). In Figure b, the dashed rectangles mark the working area, where the relative atomic motion is a remnant of electric polarization. The filament region is in the working area. (**c**, **d**) The bottom-to-top line scanning across the dashed rectangles in (**a**, **b**) for the 0 cycle (**c**) and 10^9 cycles (**d**), respectively. (**e**) Schematic of SOM device architecture with stacked OTS layers and electrodes.

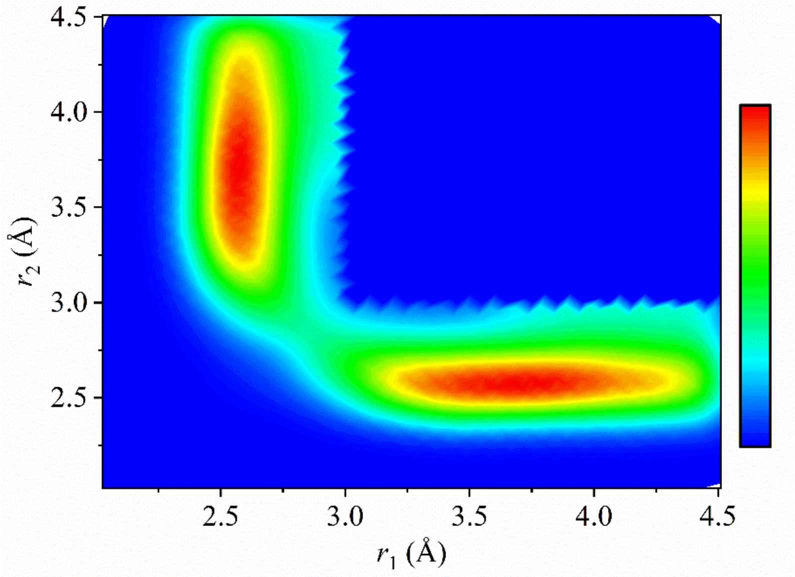

Supplementary Figure 3 Angular limited bond correlation (ALTBC) plot provides the Peierls-distortion evidence without electric field applied. It illustrates that an atom has one 2.60 Å short bond and one 3.58 Å long bond in a line. After applying electric field, the preferred Ge(bottom)-Se(up) bonds can be achieved by exchanging the long and short bonds.

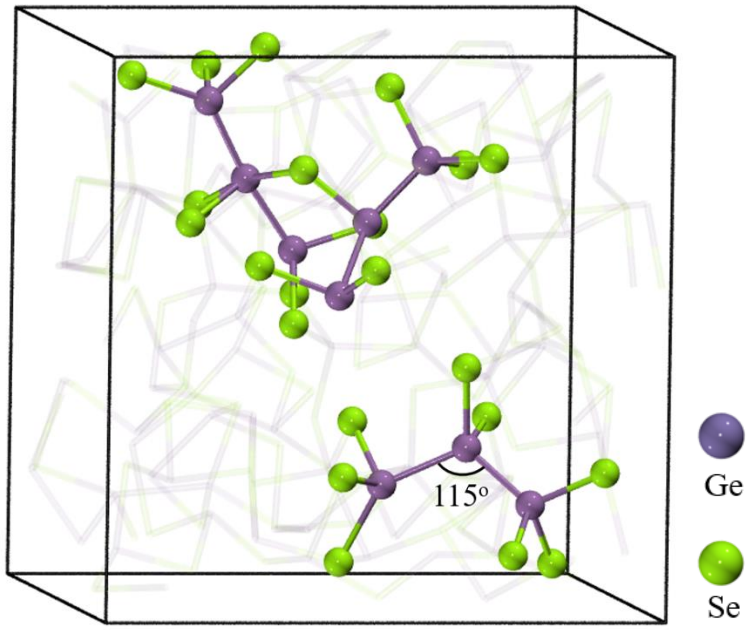

Supplementary Figure 4 Ge-Ge-Ge chains in amorphous GeSe. Bond lengths of Ge-Ge-Ge chains are approximately 2.8 Å. Top: Two Ge-Ge-Ge chains exhibit quasi-linear geometries (bond angles ~180°), generating simulated ABED patterns with dominant d-spacing of 2.8 Å. This contrasts with the experimental 1.95 Å spacing, ruling out contributions from such linear Ge-Ge chains to the observed 1.95 Å signal. Bottom: A Ge-Ge-Ge chain with a bond angle of 115° (vs. ideal sp^3 hybridization angle of 109°28'), yielding a calculated ABED *d*-spacing of 2.36 Å, derived from:

$$d = 2.8 \times sin\left(\frac{115^o}{2}\right) \text{ (Å)}.$$

The combined results demonstrate that neither linear nor distorted Ge-Ge chains can account for the experimental 1.95 Å d-spacing, suggesting alternative structural origins.

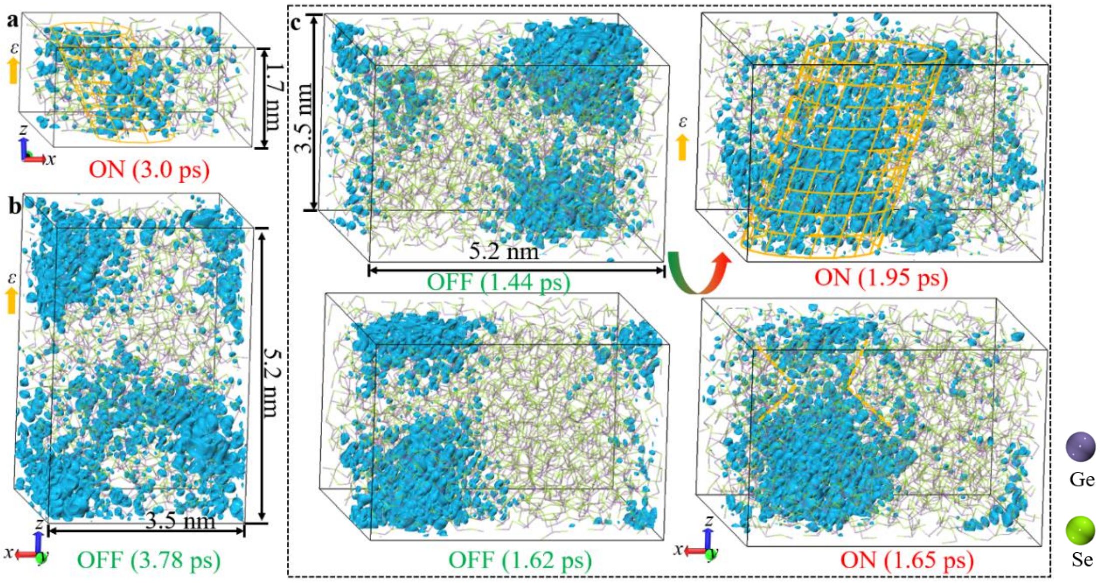

Supplementary Figure 5 Size effect on filament formation and scaling limit of devices. Atomistic view of the (**a**) 3.5 nm × 3.5 nm × 1.7 nm (800 atoms) (**b**), 3.5 nm × 3.5 nm × 5.2 nm (2,400 atoms) and (**c**) 5.2 nm × 5.2 nm × 3.5 nm (3,600 atoms) models. The system in (**a**) turns on under 0.04 V/Å fields in 3 ps with a ~2-nm filament. (**b**) When the thickness (5.2 nm) is larger than the length (3.5 nm), the system becomes harder to turn on and remains in an OFF state under a 0.06 V/Å field. (**c**) Snapshots of the filament formation and associated OFF (left) - ON (right) transition under a field of 0.06 V/Å.

We next turn to study the effect of size on the filament formation and device scaling limit using above 200-atoms model as a basic cell. For the 3.5 nm × 3.5 nm × 1.7 nm (800 atoms), a 1.4-nm diameter filament is formed at 3 ps, as shown in Figure a. However, for the 3.5 nm × 3.5 nm × 5.2 nm (2,400 atoms), the CF does not form even after a long simulation time (4.5 ps), as shown in Figure b. This is because the CF formation requires significant structural changes and cooperative rearrangement of neighboring atoms. In the experiments, pancake-shaped filaments have been observed.[1] In the 3.5 nm × 3.5 nm × 5.2 nm model, the height is 1.5 times greater than the width, making it difficult for the CF formation. For the sub-10nm scale, we design a 5.2 nm × 5.2 nm × 3.5 nm (3,600 atoms). The 3.5 nm height is chosen to avoid significant tunnel leakage currents.[2] Being larger than 3.5 nm, a 5.2 nm width enables the CF formation. Here, we suggest that scaling limit of a working SOM cell is about 5.2 nm × 5.2 nm ×

3.5 nm. Currently, the width of SOM cells in chips has reached 16 nm[3] and their capacity increases 3n times as cells are scaled down, with n being the number of stacked layers.

Figures c illustrates the process of filament formation. At 1.62 ps, two separate parts seem to form a filament. At 1.65 ps, they connect with an initially narrower filament diameter. Finally, at 1.95 ps, a filament with a diameter of ~2.4 nm is formed in the direction of the electric field. The results indicate the final filament size is dependent on the film thickness. Although the 3 × 3 × 2 model is the supercell of the 1 × 1 × 1 model, we do not observe nine isolated 1.0-nm smaller filaments but a bigger one. Before the connection, the proto-filament fragments are initially created randomly as the modelled supercell is initially homogenous. However, these fragments move to different regions of the systems between 1.44 ps and 1.62 ps, ahead of their connection after 1.65 ps.

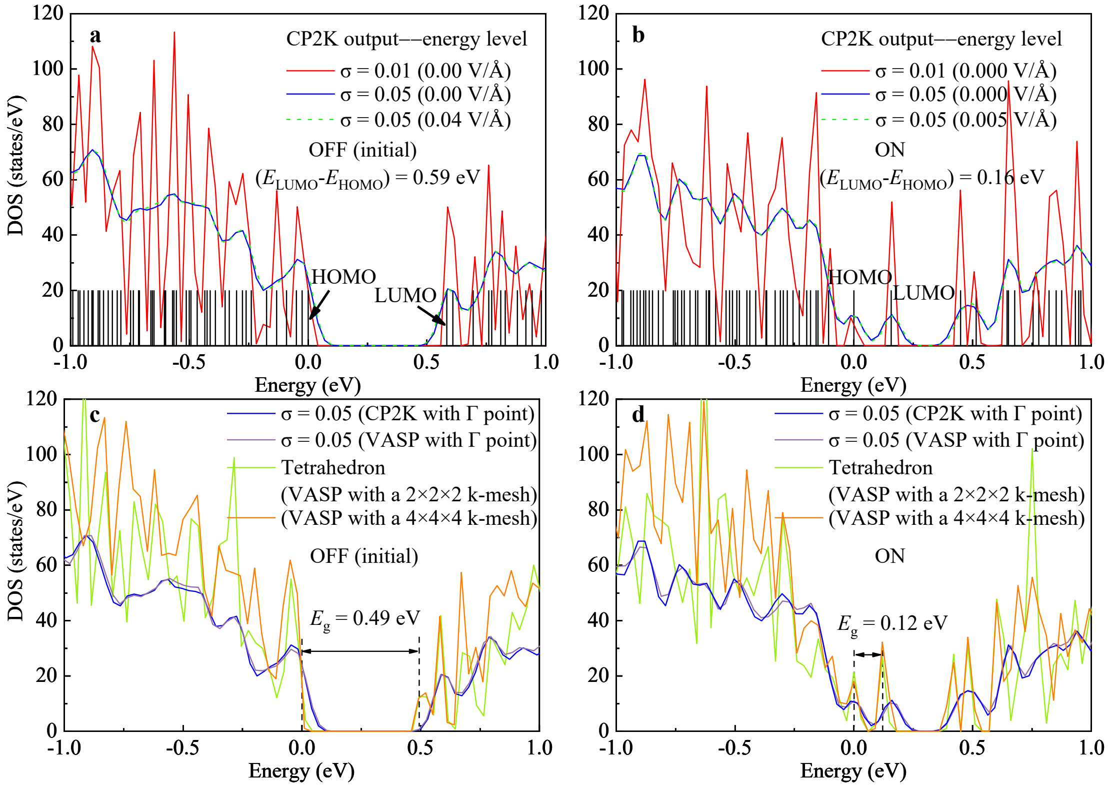

Supplementary Figure 6 DOS for the OFF (initial) state (a, c) and ON state (b, d) in the 200-atom model. Panels (a) and (b) present CP2K results: scattered and vertical lines indicate molecular orbital energy levels from CP2K output; red and blue curves represent smoothed DOS obtained using Gaussian smearing with σ = 0.01 and σ = 0.05, respectively; dashed lines show DOS under an electric field, demonstrating negligible field influence. HOMO and LUMO correspond to the zero and adjacent non-zero vertical lines, respectively. Based on CP2K energy levels, the band gaps (defined as ELUMO−EHOMO) are 0.59 eV for the OFF state and 0.16 eV for the ON state. In (c) and (d), Gaussian-broadened DOS from both CP2K and VASP almost overlap at the Γ point. Using denser k-meshes (2×2×2 and 4×4×4), the band gaps decrease to 0.49 eV (OFF) and 0.12 eV (ON), respectively. Vertical line values are multiplied by 20.

The physical bandgap (ELUMO−EHOMO) in the Γ-point condition remains finite throughout simulations, from 0.59 eV (OFF) to 0.16 eV (ON) in Figure a-b, ensuring validity of the Berry-phase polarization framework. The apparent "zero band gap" results solely from Gaussian smearing (σ=0.05 eV) during post-processing when the (ELUMO−EHOMO) value is small, a standard visualization technique for bulk materials that does not alter underlying electronic states (raw CP2K outputs show discrete energy

levels). Figure b confirms persistent bandgaps with reduced σ. In Fig. 4c of the main text, the positions of the molecular orbitals align with the vertical lines of IPR, with HOMO/LUMO positions at zero and near-zero vertical lines, respectively. On the other hand, CP2K's orbital transformation (OT) algorithm requires a finite band gap, as it ensures that wavefunction optimization via orbital rotations can proceed under integer occupations. If the system is metallic, the iteration would fail to converge. The successful simulation of threshold switching using OT algorithm confirms the absence of metallization throughout the process. Field-derived forces were thus propagated within a rigorously applicable theoretical framework.

To further verify the validation of Γ-point, we compared the DOS under various conditions, as shown in the Figure. Figures a and b demonstrate that the external electric field has minimal impact on the DOS, as the curves almost overlap with and without the field. Since the OT method in CP2K only supports Γ-point sampling and does not allow k-mesh sampling, we performed additional DOS calculations using VASP with denser k-point grids. Figures c and d reveal that the DOS calculated with Γ-point sampling in both CP2K and VASP are nearly identical, which is expected as both employed the same PBE-D3 functional and VASP used Gaussian smearing. To evaluate the effect of denser k-point sampling, we tested 2×2×2 and 4×4×4 k-meshes in VASP using the tetrahedron method to enforce integer occupations. The results show that the bandgap values at the 2×2×2 k-mesh (0.49 eV OFF and 0.12 eV ON) are slightly reduced compared to the Γ-point results (0.59 eV OFF and 0.16 eV ON), which we attribute to Brillouin zone dispersion effects. Notably, the band gap values show negligible differences between the 2×2×2 and 4×4×4 samplings, indicating that the 2×2×2 k-mesh is sufficient for convergence. The accurate calculation verifies the non-zero band gap that ensures full compliance with Berry-phase requirements. Despite of a slightly larger gap, Γ point is reasonable in our simulations.

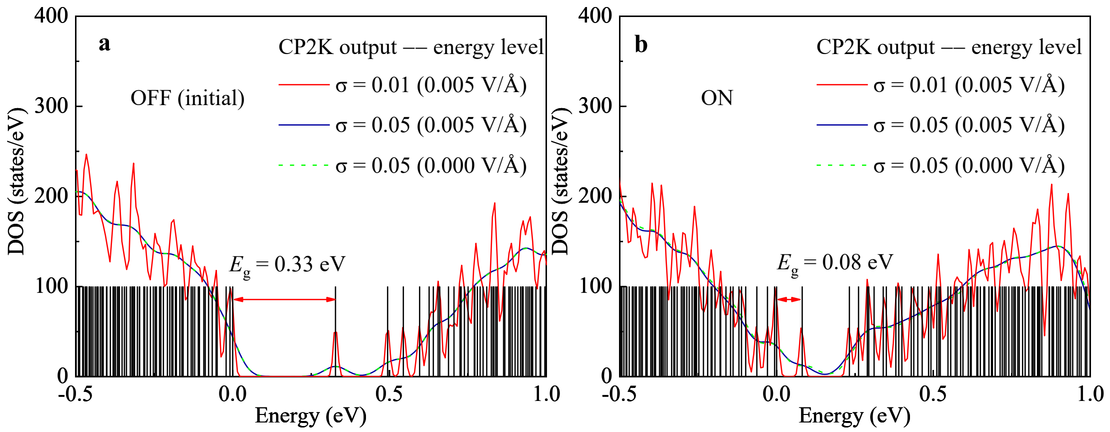

Supplementary Figure 7 DOS of the 3.5×3.5×1.7 nm^3 supercell (800 atoms) for the OFF (initial, a) and ON (b) states. The band gaps ($E_{\mathrm{LUMO}} - E_{\mathrm{HOMO}}$), obtained from the CP2K output energy level, are 0.33 eV for the initial OFF state and 0.08 eV for the ON state. Note: raw CP2K output energy level (vertical lines) scaled by 100.

Compared with the 1.7 × 1.7 × 1.7 nm^3 cubic cell with 0.057 Å^{-1} k-space resolution (Γ-point), we simulated a 3.5 × 3.5 × 1.7 nm^3 supercell achieving k-resolutions of 0.028/0.028/0.057 Å^{-1} (x/y/z). It confirms finite bandgaps at both stages: 0.33 eV (initial OFF state) and 0.08 eV (ON state). The "zero band gap" claim refers solely to Gaussian-smeared post-processing DOS (σ=0.05 eV) for conventional-bulk-material DOS visualization when the band gap is small. In fact, the raw outputs of CP2K show discrete levels, which is integer occupations. It shows that the DOSs almost overlap with and without the field. It illustrates that the external electric field has little impact on the DOS, which is similar to the 1.7 × 1.7 × 1.7 nm^3 model in Supplementary Fig. 6. Therefore, the finite gap is still persistence in the dense k-point sampling under electric field.

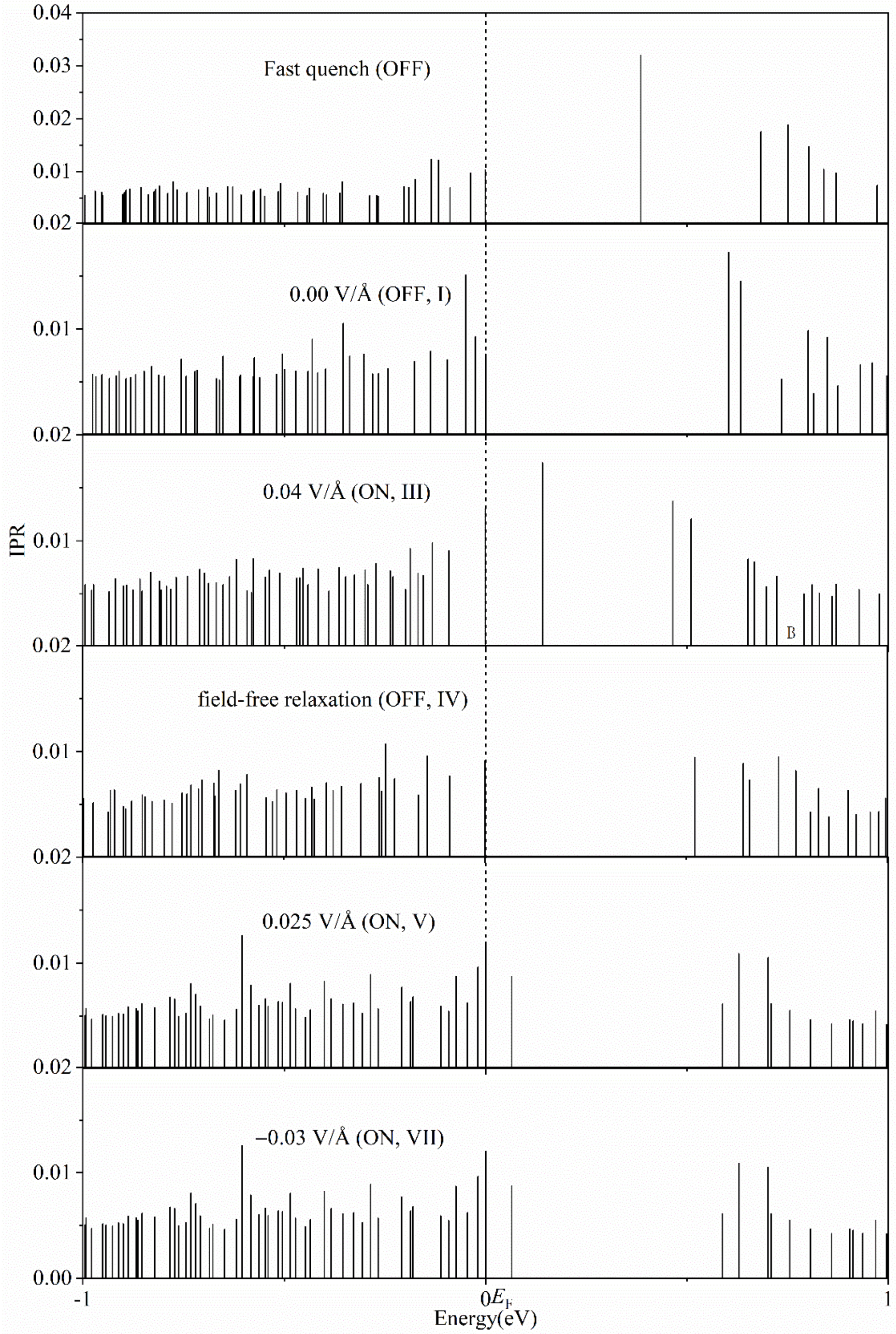

Supplementary Figure 8 IPR values across the structural evolution sequence. The IPR was calculated for the key states in the sequence: the initial fast-quench, OFF (I), ON (III), OFF (IV), ON (V), to the ON (VII) models. The OFF (IV) model serves as the initial structure for the subsequent ON (V), OFF (VI), and ON (VII) states.

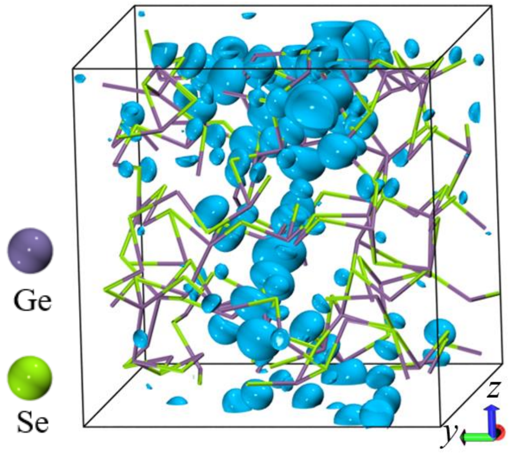

Supplementary Figure 9 The HOMO density for the turned-on (III) state after applying an electric field of 0.04V/Å. A contour plot value of 0.01 e bohr^{-3} is used. The HOMO is delocalized in a channel, overlapping with the delocalized region filled by the defect state LUMO shown in Fig. 4e.

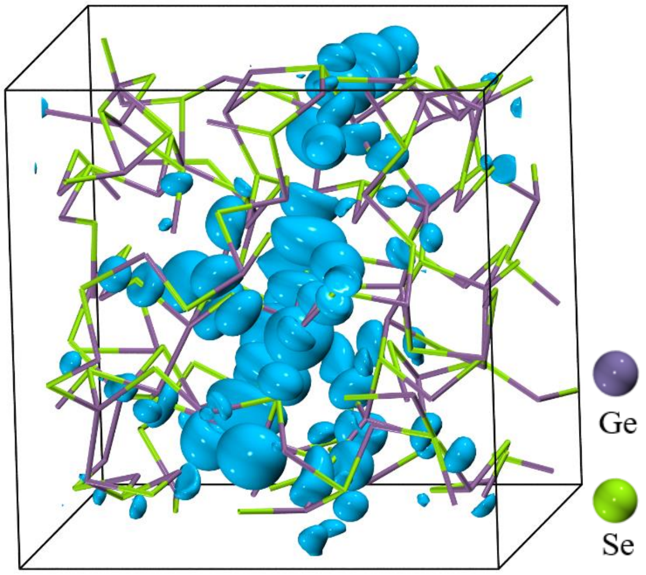

Supplementary Figure 10 Using a HSE06 hybrid functional, the electron density of the new MO (LUMO) above Fermi level for the III model. A delocalized state appears throughout the filament, which is similar to the PBE result (Fig. 4e). This confirms that the computed delocalization along the filament for the states around the Fermi level is physical and not due to the use of a semi-local XC functional (PBE as in Fig. 4e).

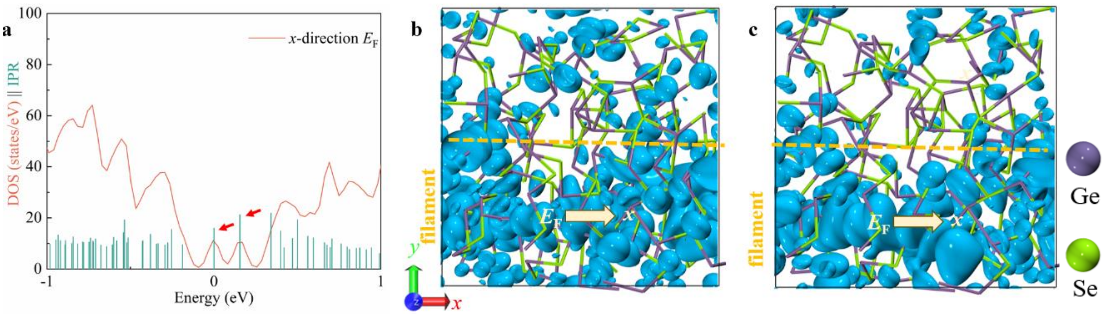

Supplementary Figure 11 Verifying that the conductive filament direction aligns with the direction of electric field at all times. (**a**) The electron DOS (red) and IPR value (blue) under the electric field along the *x* direction. (**b** and **c**) Using a contour plot value of 0.01 e bohr^{-3}, the density of the HOMO (**b**) and LUMO (**c**) states are delocalized in channels under the electric field along *x* direction. The IPR value has been multiplied by a factor 2000. The electric field applied is 0.06 V/Å.

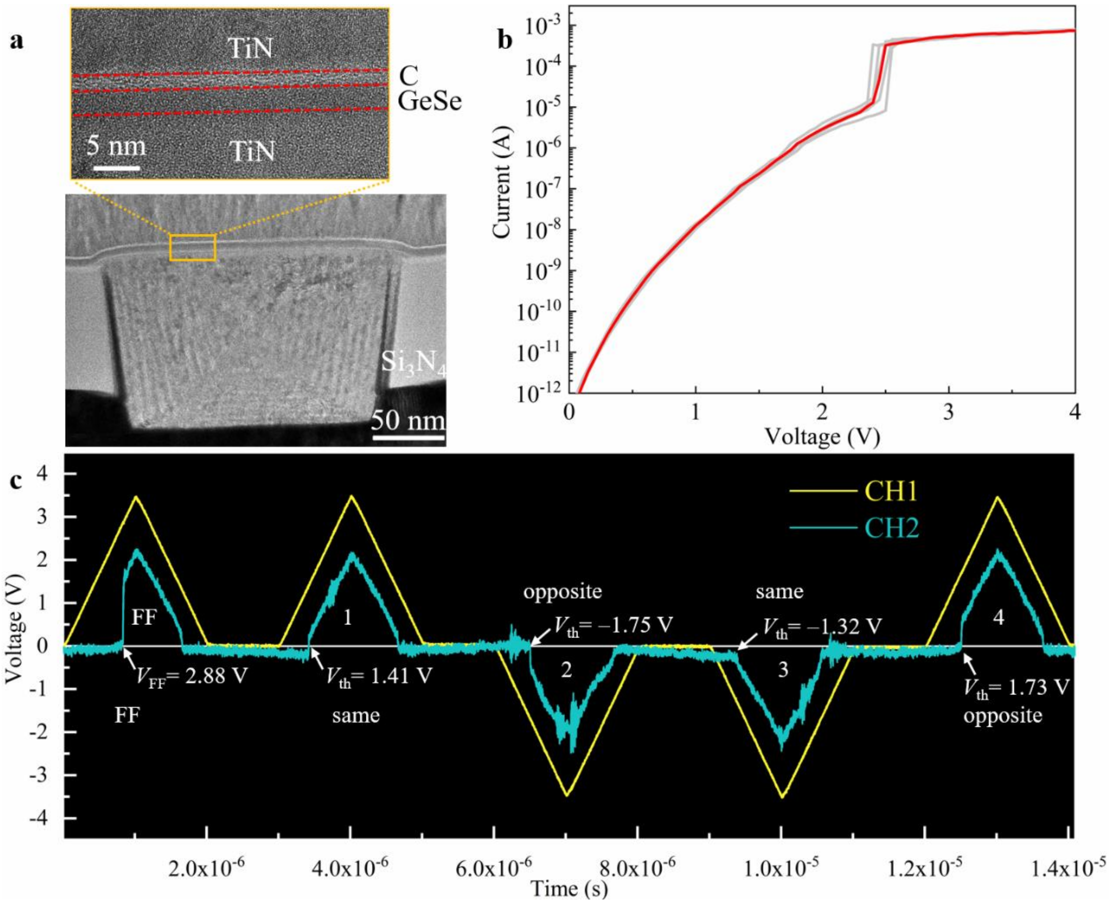

Supplementary Figure 12 Electrical characterization of 3.5-nm-thick GeSe devices. (a) Cross-sectional TEM image of a T-shaped device. The active GeSe layer is uniformly deposited at 3.5 nm thickness. (b) DC current-voltage (I-V) characteristics. (c) Pulsed I-V response under triangle pulse excitation (circuit schematic: Fig. S17). Yellow traces: Source voltage (CH1); Blue traces: Voltage (× 20) across the 100-Ω series resistor (CH2), where current (I) is derived from I = CH2/100 Ω. Note: The pulsed I-V behavior mirrors that of 20-nm-thick devices in Fig. 2f.

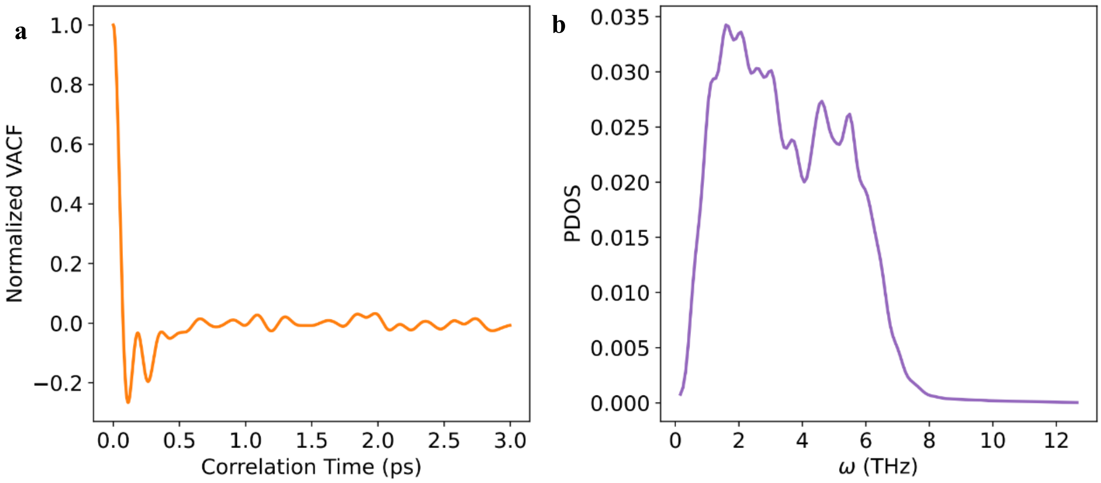

Supplementary Figure 13 Phonon density of states (PDOS) for amorphous GeSe at 300 K without electric field applied. Fourier transition of velocity autocorrelation function (VACF, **a**) is used to calculate PDOS (**b**) is calculated by using the following formula,

$$PDOS(\omega) = \int V_{ACF}(t)\, e^{-2\pi i \omega t} dt \qquad (1)$$

$$V_{ACF}(t) = \frac{< \sum_i v_i(t_0) \cdot v_i(t_0 + t) >}{< \sum_i v_i(t_0) \cdot v_i(t_0) >} \qquad (2)$$

where t_0 ant t are the initial time and correlation time, respectively. Here, we use 9-ps AIMD trajectories to calculate PDOS. The peak of low-frequency vibrations occurs around 2 THz, indicating that the typical vibration period is approximately 3.14 ps.

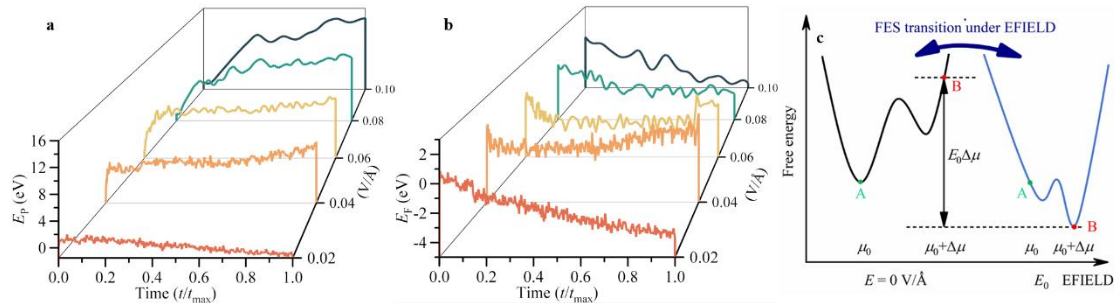

Supplementary Figure 14 Transition of free energy landscapes tuned by electric field. (**a**) The evolution of potential energy with normalized time t/t_{max}. The t_{max} values for 0.02, 0.04 (V_{FF}), 0.06, 0.08, and 0.1 V/Å are 1000 (OFF), 83.31 (ON), 9.45 (ON), 1.29 (ON), and 0.489 (ON) ps, respectively. (**b**) The evolution of free energy with normalized time, which is obtained from (a) according to formula: $E_F = E_P + W_E$. (c) A sketched diagram for the transition of free energy landscapes tuned by electric field. The left and right are the landscapes of potential energy (E = 0) and free energy (E = E_0), respectively.

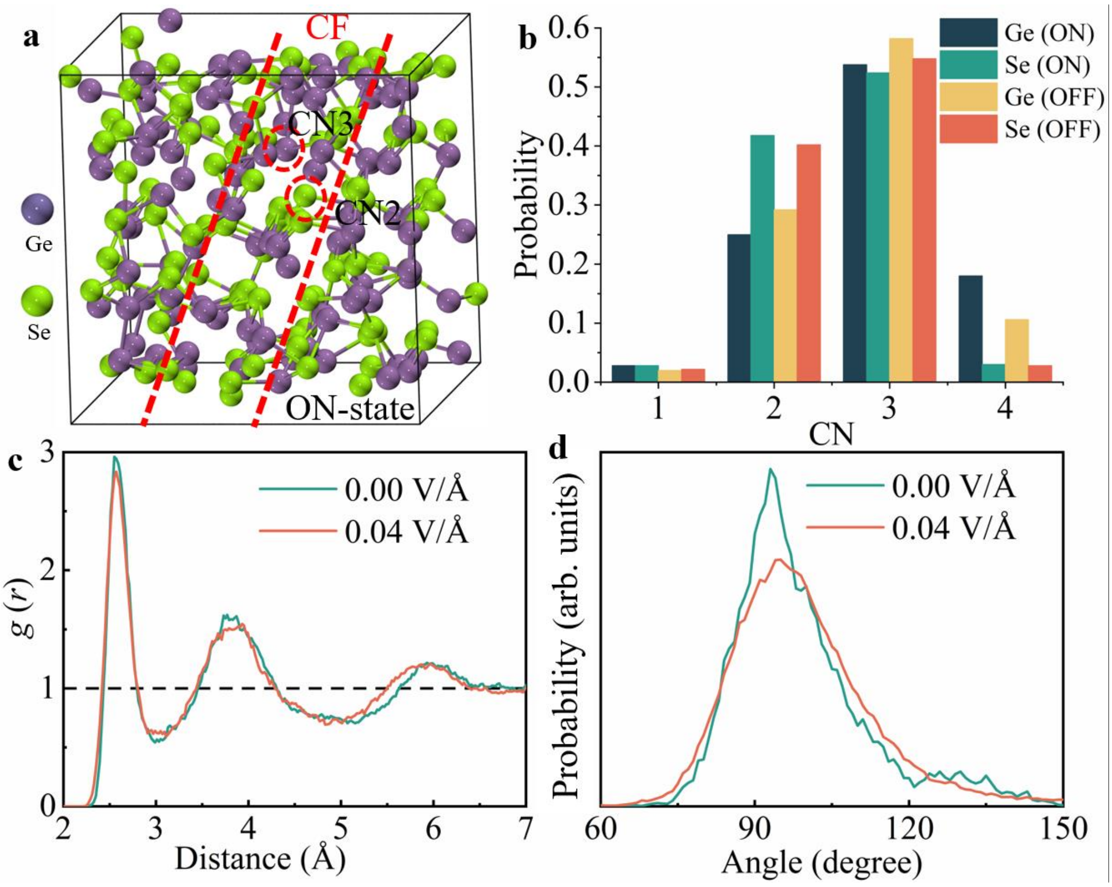

Supplementary Figure 15 The structural comparison between the 0V/Å-OFF and 0.04V/Å-turn-on states. (a) The amorphous structure of the ON-state at 0.04V/Å (Fig. 4d), whose coordination number (CN) is similar to the OFF state. (**b**) shows similar CN distributions for the turn-on and OFF states. (**c**) and (**d**) show the difference in both pair distribution function (PDF) and bond angle by comparing the 0V/Å- and 0.04V/Å-operated states. Compared with the 0V/Å structure, the 0.04V/Å structure has slight longer bonds, illustrated by the lower first peak and higher first valley of PDF. The distribution of bond angle at 0.04 V/Å is wider than the 0 V/Å. Therefore, it illustrates more structure distortion at high-energy turn-on states. The reduced bandgap or newly formed MO above HOMO originates from the structure distortion, particularly in the channel with more structural change.

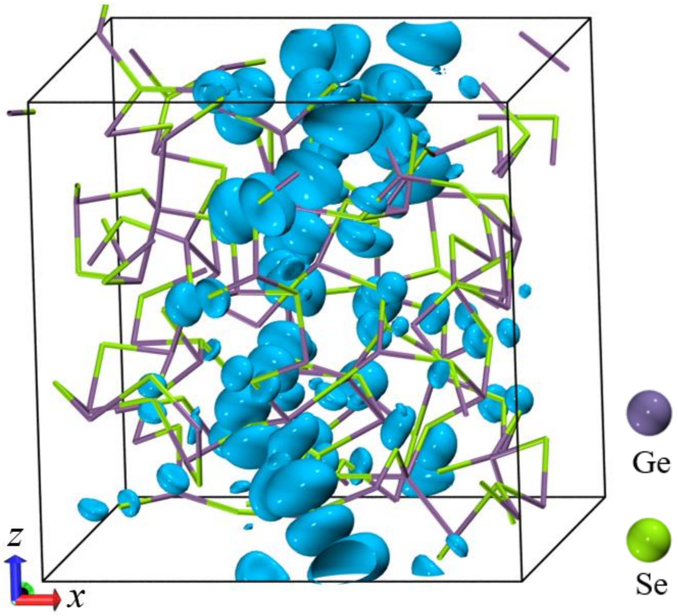

Supplementary Figure 16 Observing a filament at 50 K under 0.08 V/Å along the *z*-axis. A contour plot value of 0.01 e bohr^{-3} is used. The *NVE* ensemble is used and temperature increases from 50 K to 274 K. For the other 1 × 1 × 1 (0.04 V/Å), 2 × 2 × 1 (0.06 V/Å)), and 3 × 3 × 2 (0.06 V/Å) models, the temperature increases from 300 K to 401 K, 557 K, and 593 K, respectively.

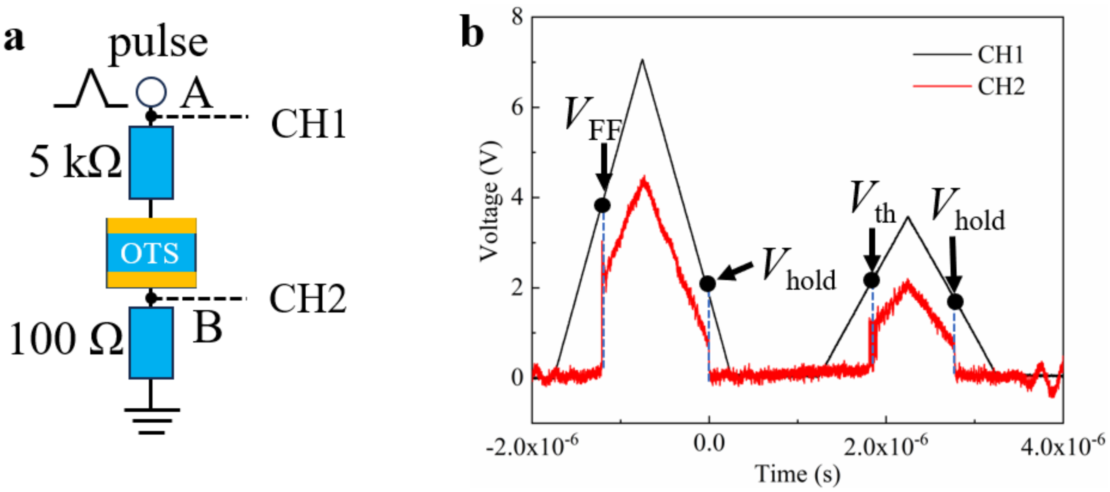

Supplementary Figure 17 Circuit diagram for device testing. (**a**) Circuit diagram for device testing. A 5kΩ series resistor is used to limit the turn-on current. The electric potentials of channel-1 (CH1) at A point and channel-2 (CH2) at B point, monitored by oscilloscope, instantaneously display the practical output voltage of power supply and the voltage across the 100Ω series resistor, respectively. (**b**) The electric potentials monitored in CH1 and CH2 were recorded after generating two triangle pulses, with the CH2 value multiplied by 30 herein. The current in the circuit is equal to the CH2 voltage divided by 100 Ω. The voltage across OTS is equal to the CH1 voltage minus the voltages across 5 kΩ and 100 Ω resistors. Then, the I-V characteristics of OTS are obtained. On the other hand, the V_{FF}, V_{th}, and V_{hold} values are shown in (**b**).

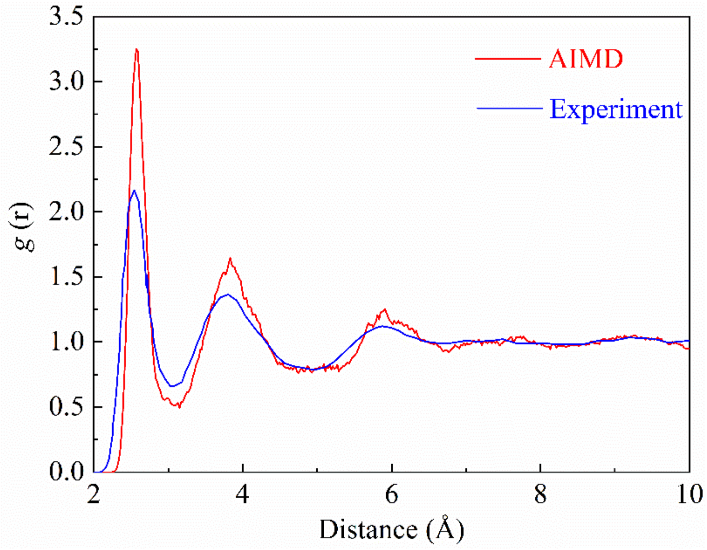

Supplementary Figure 18 Comparison of the experimental and simulated pair distribution functions in amorphous GeSe at 1000 K. The two curves are similar. The peak position from AIMD shift slightly towards larger values compared to the experiment. It is because the generalized gradient approximation (GGA) functional used here always overestimates bond length slightly compared to the experimental value, a phenomenon observed in other amorphous systems as well[4]. The experiment value is obtained from the reference.[5]

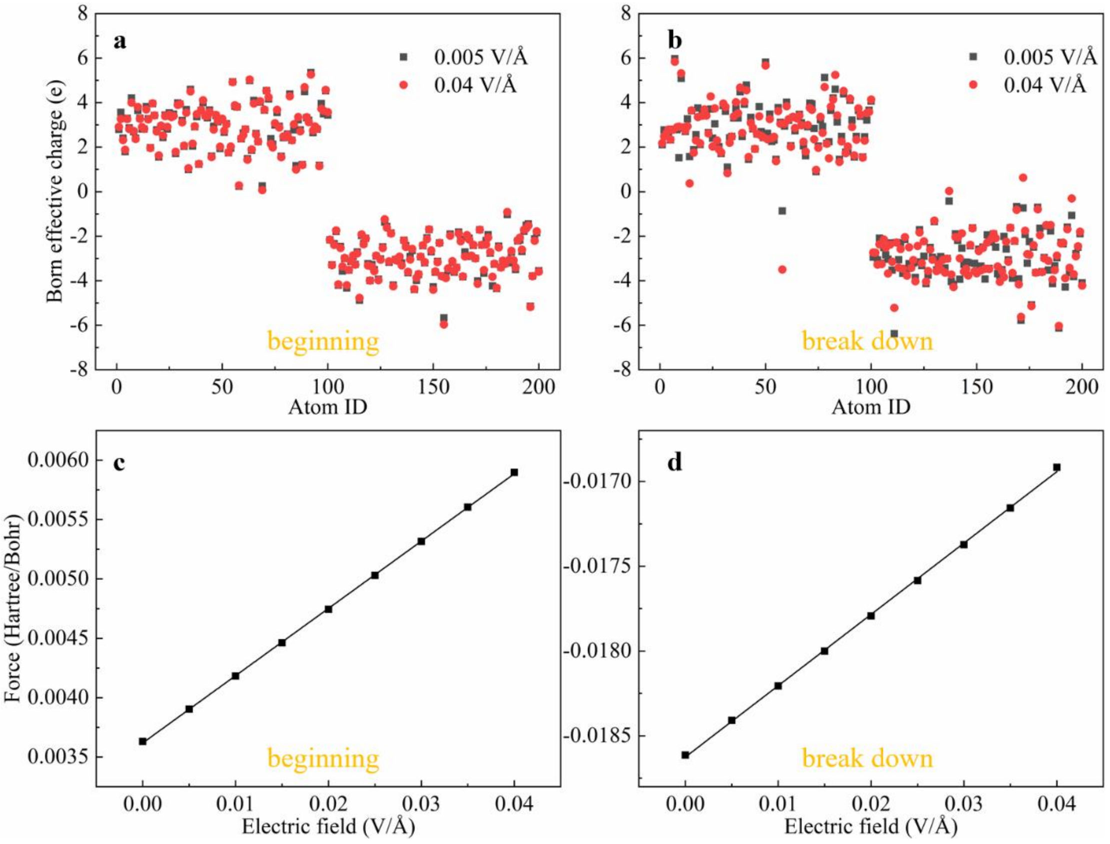

Supplementary Figure 19 Verifying the rationality of obtaining high-field force from the low field condition. Comparison of the Born effective charges between the 0.005 V/Å and 0.04 V/Å testing conditions at the initial (**a**) and break down (**b**) points. The change of the *z*-axis force for one atom as the electric field changes from 0.0 V/Å to 0.04 V/Å at the initial (**c**) and break down (**d**) points. The break down is caused by non-convergence of the electronic self-consistent procedure, which always occurs on the system with small bandgap for the Orbital Transformation (OT) algorithm used. The OT algorithm, which directly optimizes wavefunctions through orbital rotations, is efficient for systems with a band gap due to its reliance on preconditioning the occupied orbitals, but it struggles with gapless systems where the lack of a clear energy separation hinders convergence. At the initial time, the Born effective charges are almost the same under the two field strength conditions. On the other hand, at the breakdown point, a few atoms present larger charge difference while results for the other atoms are similar. Both at the initial and break down points, a linear change of the *z*-axis force is present as the field changes from 0.0 V/Å to 0.04 V/Å. These results justify the rationale for predicting electric forces using a linear extrapolation strategy in the simulations.

Figures a and b show large Born effective charges in amorphous chalcogenides; meanwhile, the covalent bond is weak in amorphous chalcogenides. The bond energy is 37.6 kcal/mol for Ge–Ge, 30.2 kcal/mol for Sb–Sb, 33 kcal/mol for Te–Te, 34 kcal/mol for Ge–Sb, 37.9 kcal/mol for Ge–Te, 32.8 kcal/mol for Sb–Te, 44 kcal/mol for Se–Se, 50.9 kcal/mol for S–S, 51.5 kcal/mol for Ge–Se, 44 kcal/mol for Sb–Se, 58.4 kcal/mol for Ge–S, 50 kcal/mol for Sb–S, 32.1 kcal/mol for As–As, 43.0 kcal/mol for As–Se and 88.2 kcal/mol for Si–O. Therefore, amorphous chalcogenides display significant electric responses.

Supplementary References

1 Petersen, K. E. & Adler, D. On state of amorphous threshold switches. *Journal of Applied Physics* **47**, 256-263 (1976).

2 Frank, D. J. *et al.* Device scaling limits of Si MOSFETs and their application dependencies. *Proceedings of the IEEE* **89**, 259-288 (2001).

3 Park, I. M. *et al.* in *2023 International Electron Devices Meeting (IEDM).* 1-4.

4 Zhu, M. *et al.* Direct atomic insight into the role of dopants in phase-change materials. *Nat. Commun.* **10**, 3525 (2019).

5 Salmon, P. S. Structure of liquids and glasses in the Ge–Se binary system. *J. Non-Cryst. Solids* **353**, 2959-2974 (2007).
