# Supplementary material for: Non-Arrhenius threshold switching by field-driven dipolar ordering
Source: Nat Commun. 2026 May 13;17:6404. doi: 10.1038/s41467-026-72970-z (PMC13376810; doi:10.1038/s41467-026-72970-z)
Supplement: Supplementary file 2 — Transparent Peer Review file [file 41467_2026_72970_MOESM2_ESM.pdf]

nature portfolio

Peer Review File

Non-Arrhenius threshold switching by field-driven dipolar ordering

Corresponding Author: Professor Yan Cheng

This manuscript has been previously reviewed at another journal. This document only contains information relating to versions considered at Nature Communications.

This file contains all reviewer reports in order by version, followed by all author rebuttals in order by version.

Version 0:

Reviewer comments:

Reviewer #1

(Remarks to the Author)
In the revised manuscript, the authors have addressed some of my previous concerns by expanding the k-point sampling and increasing the supercell sizes. However, I do not believe the computational study adequately explains the origin of threshold switching.

First, consider the experimental data in Fig. 2e (the red data points after FF). By "threshold switching," we refer to a behavior in which, for example, the film is non-conducting at 1 V but becomes conducting at around 3 V. The mechanism of threshold switching should account for why such a transition occurs across the threshold voltage (Vth).

Now, turning to the computational results in Fig. 4b and 4c: the authors show the formation of a conductive filament (CF) at 0.04 V/Å, which is then maintained at 0.02 V/Å. This does NOT represent threshold switching. To demonstrate threshold switching, after CF formation, the film should revert to an insulating state at a lower bias, for instance, at +0.01 V/Å.

Furthermore, additional tests are required to fully confirm the SOM behavior reported in Fig. 2f. For example, after applying – 0.03 V/Å (step VI in Fig. 4b), a subsequent positive bias scan should be performed to verify whether Vth shifts to higher values, as observed experimentally in Fig. 2f.

These analyses and discussions are missing from the current manuscript. The presented simulations mainly describe CF formation, which is more closely related to the "forming" process (FF) observed in the experiments, rather than threshold switching itself.

Therefore, I conclude that the MD simulations are only indirectly related to the threshold switching and SOM characteristics. I do not recommend publication of this work in its current form.

(Remarks on code availability)

Reviewer #2

(Remarks to the Author)
This manuscript reports on a combined computational and experimental investigation of the switching threshold mechanism in amorphous chalcogenides. This mechanism consists of an abrupt increase in conductivity at a critical threshold voltage and is exploited in the selector elements of phase-change random-access memories (PCRAMs). Moreover, it plays a key role in selector-only memories, which achieve faster switching speeds and lower power consumption than conventional phase-change memories, at the expense of losing non-volatility.

The physical mechanism responsible for threshold switching has been debated for several decades. In particular, both electronically driven and structurally driven models have been proposed. The main claim of the paper is that threshold

switching in GeSe—a prototypical selector material—stems from dipolar ordering of atoms induced by the external electric field. This ordering, in turn, guides the formation of conductive filaments oriented along the electric field. The authors also argue that this mechanism can explain the threshold voltage asymmetry in selector-only memories as well. These claims are supported by atomic-resolution angstrom-beam electron diffraction and ab initio molecular dynamics simulations performed under an applied electric field.

The topic of the paper is certainly interesting and important, and the manuscript is well written. It has already undergone a first round of review. While the second reviewer provided a positive assessment, the first referee raised serious concerns about the validity of the simulations, noting that the bulk dipole moment can be computed within the Berry-phase approach only in systems with a finite electronic energy gap. I fully agree with this point. Apparently, this comment was triggered by an incorrect statement in the first version of the manuscript, namely that the ON state is a zero-gap state. The authors have clarified that, in fact, this state exhibits a non-negligible energy gap, and that the gap never closes during the ab initio simulations. I find their reply convincing, as they now provide computational evidence for the presence of a finite HOMO-LUMO gap in the new figures included in the revised manuscript and in the Supplementary Information. Nevertheless, the authors should clarify the following points before I can recommend publication of this work in Nature Communications.

I agree that monitoring the gap between the HOMO and LUMO states is essential to assess the validity of the Berry-phase approach. However, based on Figure 4c, one could argue that the “band gap”—or, more precisely, the (Tauc) optical gap—remains on the order of 0.5 eV throughout the simulation, and that the states appearing within this gap correspond to midgap or defect states. Typically, these states are strongly localized, hence I am somewhat puzzled by the fact that in your "ON" models they seem to be extended. The authors should comment on this point. The charge-density isosurfaces shown in Figures 4e–g do not provide sufficient evidence for delocalization, since one can always produce a delocalized isosurface by tuning the isovalue. Computing the IPR values is the correct approach. The authors did compute this quantity; however, it is not possible to compare the IPR values of these delocalized states with those of the other relevant states (including the defect state in the “OFF” model) in Figure 4c because the plots are too small. Even after zooming in, it is not clear whether the IPR value of the LUMO state in the “ON” model is significantly lower than that of the LUMO state in the “OFF” model. The authors should include additional plots of DOS and IPR versus energy (similar to Figure S10a) for several “OFF” and “ON” models in the main text to facilitate comparison between the IPR values.

Furthermore, based on Figure S10 (which shows results for an electric field aligned along the x direction), one could argue that the LUMO state appears more localized than the states deep in the valence and conduction “bands,” apparently contradicting the claims of the paper. Nevertheless, a relatively large IPR value in a small supercell does not necessarily imply strong localization in all directions; it could also reflect a quasi-1D or quasi-2D character of the electronic state (this point has been discussed in previous computational works on the electronic properties of PCMs).

Finally, I am wondering why the authors do not observe strongly localized states around Ge–Ge homopolar bonds or other defects in the ON models, but only in the fast-quenched OFF model. While I understand that fast-quenched models contain a higher concentration of Ge–Ge bonds, I would still expect such states to appear in at least some ON models. The authors should briefly comment on this point.

(Remarks on code availability)

Reviewer #3

(Remarks to the Author)
This manuscript presents a groundbreaking study that successfully resolves a long-standing challenge in the field of amorphous chalcogenides: the atomic-scale mechanism of threshold switching (TS). By synergistically combining state-of-the-art experimental techniques (Angstrom-Beam Electron Diffraction, ABED) and advanced computational methods (Electric-Field-coupled Ab Initio Molecular Dynamics, EF-MD), the authors provide compelling evidence for a field-driven dipolar ordering paradigm. This work shifts the understanding of TS from purely electronic or slow structural models to a unified structural-electronic mechanism governed by ultrafast, non-Arrhenius dipolar reconfiguration. The findings are of fundamental importance and have significant implications for the design of next-generation ultrafast, low-power memory technologies.

The primary weaknesses raised by the previous reviewers, concerning the computational framework's validity and the clarity of experimental methods, have been extensively and successfully addressed in the revision and rebuttal. The added methodological details and supplementary data significantly strengthen the manuscript. While the 3600-atom model is impressive and shown to be representative, it still represents a simplified, defect-free region of the amorphous material. Real devices may contain a broader distribution of intrinsic defects and stoichiometric variations that could influence the statistical formation of filaments. The authors acknowledge this through their discussion of stochastic branching at topological defects. The proposed mechanism is meticulously demonstrated for amorphous GeSe. While it is highly plausible that similar dipolar effects exist in other chalcogenides (e.g., Ge-Sb-Te, As-Se systems), direct experimental and computational evidence for other compositions is outside the scope of this paper. The generality of the paradigm remains to be fully tested across the material class.

The dielectric spectroscopy (Fig. 5) shows a significant dipole polarization response. Could the authors comment on how the measured relaxation times of these dipoles relate to the picosecond switching times observed in EF-MD? Is the

measured response primarily from the bulk matrix, while the switching involves a more correlated, sub-ensemble of "active" dipoles?

In the hierarchical CF growth model, the dipolar chains form first, and the CF grows perpendicularly. What is the precise electronic structure at the interface between a dipolar chain and the nascent CF? Is the chain itself conductive, or does it merely create a localized strain or potential field that guides the nucleation of a metallic filament?

Finally, this manuscript represents a seminal contribution to the fields of phase-change materials, non-volatile memory, and the physics of disordered systems. The work is exceptionally well-executed, the evidence is robust and multi-faceted, and the findings are of the highest significance. The authors have satisfactorily resolved all major criticisms. I strongly recommend acceptance of this manuscript for publication after minor revision.

(Remarks on code availability)

Version 1:

Reviewer comments:

Reviewer #1

(Remarks to the Author)
I appreciate the authors' comprehensive efforts in demonstrating the complete "OFF-ON-OFF-ON" switching cycle via field-free relaxation, which confirms the recovery of the insulating state. The computational validation of threshold voltage asymmetry now provides a physical explanation for the state-dependent memory behavior observed in SOM devices. I feel that these revisions successfully bridge the gap between atomistic simulations and experimental observations by establishing a unified paradigm for field-driven dipolar ordering in amorphous chalcogenides. I am satisfied with the technical clarifications provided.

Upon the request from the Editor, I have also examined the responses to the Reviewer #3's comments. The first question on the relation between relaxation time and switching time was answered satisfactorily and the modification to the manuscript is also reasonable. While the rebuttal to the second comment is reasonable, the manuscript modification is rather short. The Reviewer questioned on the precise electronic structure at the interface between the dipolar chain and the nascent CF, specifically asking whether the chain itself is conductive or merely provides a localized strain/potential field. In the rebuttal letter, the authors provided a highly convincing physical explanation, but the single sentence added to the revised manuscript is too brief. I recommend that the authors incorporate a concise version of their rebuttal explanation directly into the main text. Once this minor addition is made, I believe the manuscript will be fully ready for publication.

(Remarks on code availability)

Reviewer #2

(Remarks to the Author)
The authors have addressed my comments and concerns in a satisfactory way, and I recommend publication of the manuscript in its present form.

I only have one final remark. In their response, the authors state that

"Crucially, both the HOMO and LUMO states, with an IPR of ~0.01, fall well within the established range for delocalized states according to the literature (Nat. Commun. 10, 3065 (2019)), which defines delocalized states as having IPR values between 0.01 and 0.02."

However, the range of IPR values associated with delocalized states depends on the system size: the larger the system, the smaller the IPR of extended states. In the thermodynamic limit, the IPR of a delocalized state vanishes. Since the system size considered in Nat. Commun. 10, 3065 (2019) differs from that of the models used here, the range of values reported in that study cannot be used to discriminate between localized and delocalized states in this work.

(Remarks on code availability)

Open Access This Peer Review File is licensed under a Creative Commons Attribution 4.0 International License, which permits use, sharing, adaptation, distribution and reproduction in any medium or format, as long as you give appropriate credit to the original author(s) and the source, provide a link to the Creative Commons license, and indicate if changes were made.
In cases where reviewers are anonymous, credit should be given to 'Anonymous Referee' and the source.
The images or other third party material in this Peer Review File are included in the article’s Creative Commons license, unless indicated otherwise in a credit line to the material. If material is not included in the article’s Creative Commons license and your intended use is not permitted by statutory regulation or exceeds the permitted use, you will need to obtain permission directly from the copyright holder.
To view a copy of this license, visit https://creativecommons.org/licenses/by/4.0/

Reviewer #1:

In the revised manuscript, the authors have addressed some of my previous concerns by expanding the k-point sampling and increasing the supercell sizes. However, I do not believe the computational study adequately explains the origin of threshold switching.

Reply: We appreciate the reviewer who acknowledged our efforts in revising the manuscript, including the expanded k-point sampling and larger supercell sizes. In the following response, we elaborate on other specific technical details in question to clarify the reviewer's concerns.

Q1: First, consider the experimental data in Fig. 2e (the red data points after FF). By "threshold switching," we refer to a behavior in which, for example, the film is non-conducting at 1 V but becomes conducting at around 3 V. The mechanism of threshold switching should account for why such a transition occurs across the threshold voltage (Vth).

Now, turning to the computational results in Fig. 4b and 4c: the authors show the formation of a conductive filament (CF) at 0.04 V/Å, which is then maintained at 0.02 V/Å. This does NOT represent threshold switching. To demonstrate threshold switching, after CF formation, the film should revert to an insulating state at a lower bias, for instance, at +0.01 V/Å.

Reply: We thank the reviewer for this critical comment. We agree that a complete demonstration of threshold switching must include the reversible transition to the insulating state. We confirm that our simulations indeed capture this "OFF-ON-OFF-ON" full cycle, and that we did not miss the second insulating state.

In our EF-MD simulations, the initial formation of the CF occurs at a higher field strength of 0.04 V/Å (V_{FF}), which represents the "switching-ON" event. Fig. R1a shows the initial OFF (I) state that becomes the ON state under the 0.04 V/Å, with the reduced band gap in Fig. R1b (III) and the visual filament formation in Fig. 4e of the main text. Importantly, after this formation, we explicitly investigated the state retention. We

found that sustaining the ON state requires a holding voltage (V_{hold}) of 0.005 V/Å. Crucially, when the electric field is completely removed (0 V/Å) for a 4.5 ps MD relaxation, the system reliably reverts to an insulating OFF state (Step IV). The DOS of this relaxed OFF state (provided in Fig. R1c) confirms that the band gap recovers to a value like that of the initial OFF state. This OFF state then served as a new initial structure for the subsequent switching cycles, for example, ON again (Step V) at a lower V_{th}, as shown in Fig. R1d. Therefore, the film indeed reverts to an insulating state at a bias lower than +0.005 V/Å, fulfilling the criterion for threshold switching that the reviewer correctly emphasized. We have revised the relevant section in the manuscript to more clearly articulate this complete OFF-ON-OFF cycle and the role of the holding voltage, ensuring it aligns with the experimental observation of a higher initial forming voltage (V_{FF} ≈ 4 V) followed by a lower threshold voltage (V_{th} ≈ 2.3 V).

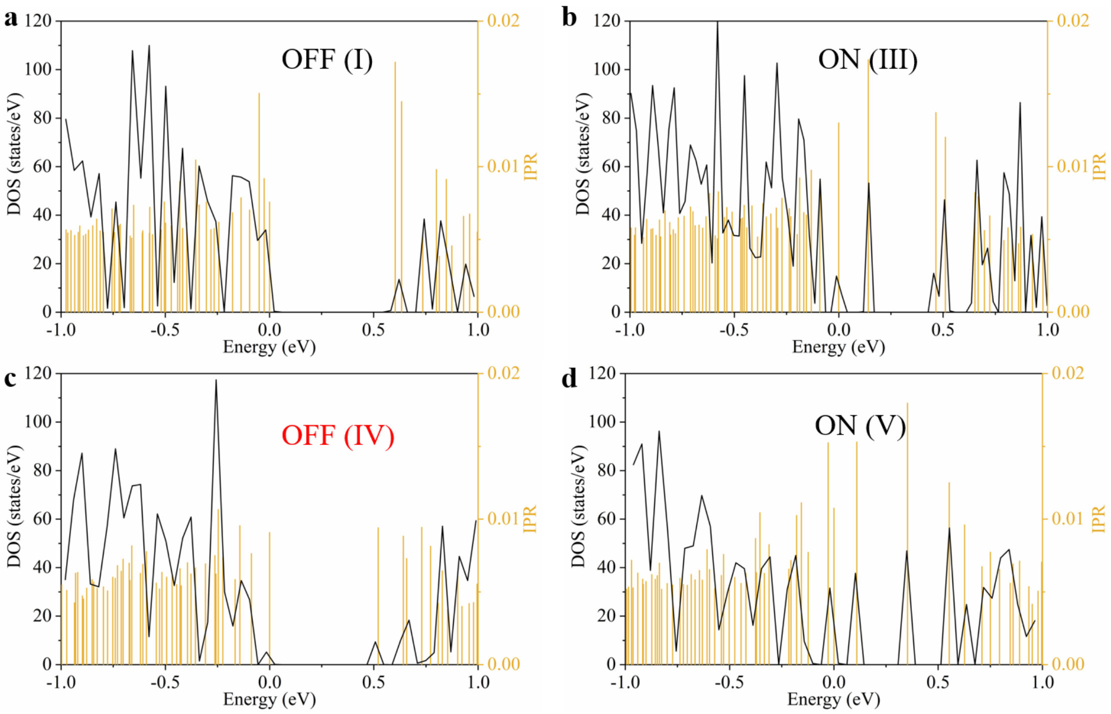

Fig. R1 Density of states (DOS) for the four structures representing a complete "OFF-ON-**OFF**-ON" switching cycle. **(a)** Initial OFF state (Step I), obtained via melt-quenching at 10 K/ps. **(b)** First ON state (Step III), simulated under an electric field of 0.04 V/Å. **(c) Second OFF state (Step IV)**, obtained after 4.5 ps of field-free MD relaxation at 300 K starting from the ON state in **(b)**. **(d)** Second ON state (Step V), simulated under a reduced electric field of 0.025 V/Å and started from OFF state in **(c)**. The DOS curves are Gaussian-broadened (σ = 0.01 eV) projections of the discrete molecular orbitals.

To make this clear, we make the corresponding changes on **page 10**. We add an OFF state (IV) after field-free MD relaxation, whose DOS is shown in Fig. R1c, to the sequence of OFF-ON-OFF-ON transition. The state IV is added into the schematic diagram of pulse operations in Fig. R2a (Fig. 4b in the main text). We also add its DOS and IPR curves in Fig. R2b (Fig. 4c in the main text). Note that both states IV and VIII are OFF states obtained from free-field MD relaxation; state IV was not exhibited in the previous version. We revise the following sentences: "Sustaining the ON-state requires a holding voltage V_{hold}=0.005V/Å. After 4.5 ps MD-relaxation without E-field (Step IV), the subsequent switching-ON occurs at V_{th}=0.025 V/Å (state V). Strikingly, negative polarity demands a higher threshold $|V_{th}|$=0.03V/Å (Fig. 4b) (state VII)."

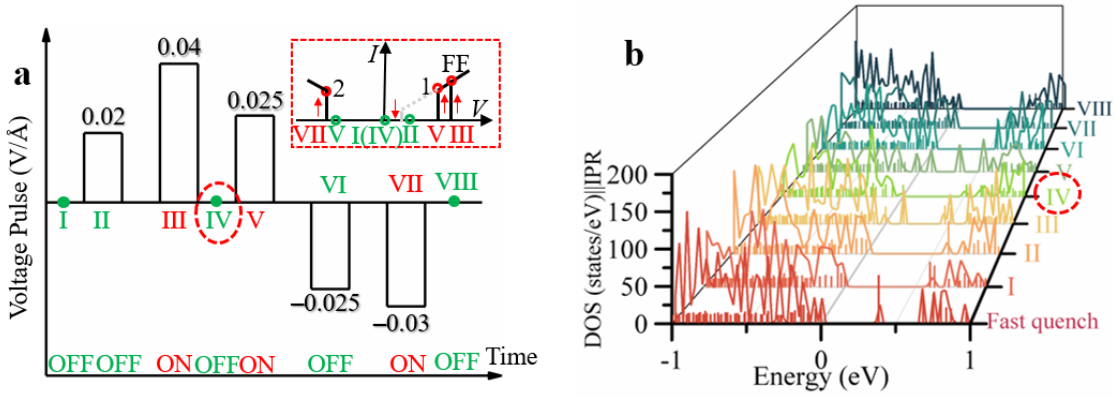

Fig. R2 (a): The schematic diagram for pulse operations. **(b)**: DOS for eight metastable configurations (labeled I-VIII) post-pulse operation, compared to a fast-quenched reference. All DOSs represent Gaussian-broadened (σ = 0.01 eV) projections of discrete molecular orbitals.

Q2: Furthermore, additional tests are required to fully confirm the SOM behavior reported in Fig. 2f. For example, after applying –0.03 V/Å (step VI in Fig. 4b), a subsequent positive bias scan should be performed to verify whether Vth shifts to higher values, as observed experimentally in Fig. 2f.

These analyses and discussions are missing from the current manuscript. The presented simulations mainly describe CF formation, which is more closely related to the "forming" process (FF) observed in the experiments, rather than threshold switching itself.

Reply: We thank the reviewer for this suggestion. To confirm the V_{th} shift, a key

feature of SOM, we compared the threshold voltages under opposite polarities. As show in Fig. R3, our simulations show that |V_{th}(negative)| (–0.03 V/Å) is indeed greater than |V_{th}(positive)| (+0.025 V/Å), directly demonstrating the V_{th} shift observed in Fig. 2f. Thus, the protocol we implemented (comparing Steps V and VII) serves as a computational validation of the SOM behavior.

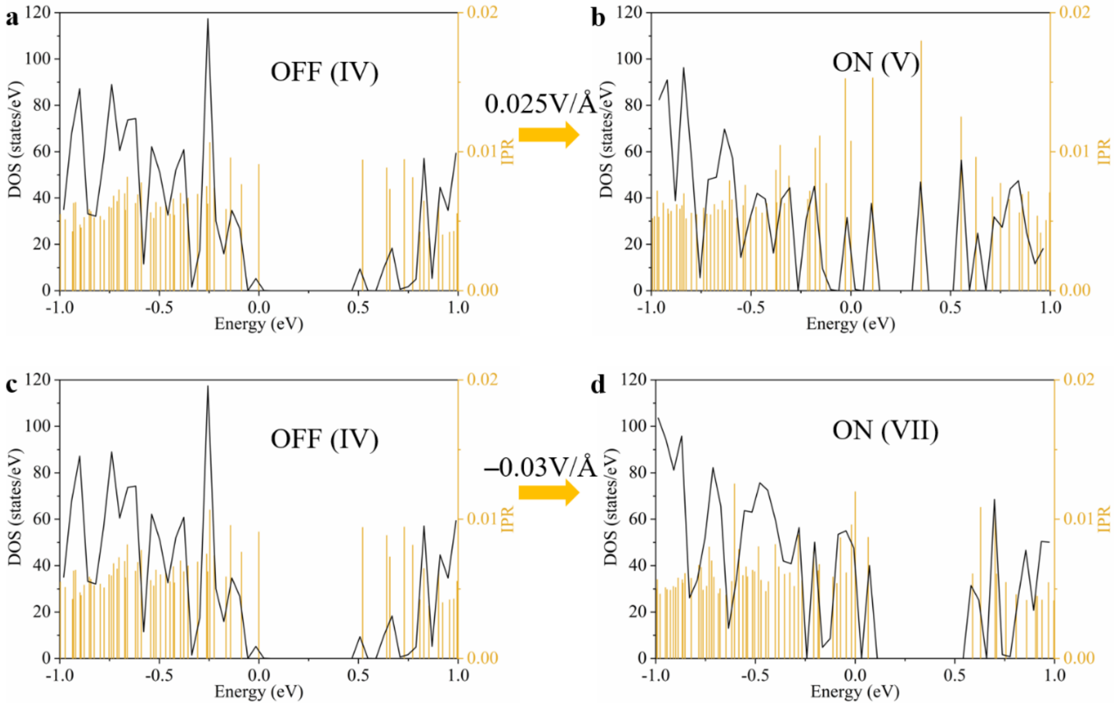

Fig. R3 We show the OFF (IV) to the ON (V) transition under positive 0.025V/Å electric field and the OFF (IV) to the ON (VII) transition under negative 0.03V/Å electric field. It shows the polarity-dependent V_{th} shift used in SOM.

In our study, the core manifestation of the SOM behavior is the directional dependence of V_{th}, which is a direct result of the non-volatile dipole orientation after the initial forming process. Our simulations explicitly demonstrate this asymmetry, as shown in Fig. R3. Starting from the same OFF state (the relaxed structure after the initial ON state at +0.04 V/Å, Step IV), the threshold switching occurs at V_{th} = +0.025 V/Å under a positive bias (Step V), namely the switching process from Fig. R3a to Fig. R3b. In contrast, when a negative bias is applied, the device does not turn on at the symmetrical field strength of –0.025 V/Å (Step VI). A higher magnitude of V_{th} = –0.03 V/Å (Step VII) is required to achieve the ON state, namely from Fig. R3c to Fig. R3d. This |V_{th}(negative)| > |V_{th}(positive)| relationship is the computational evidence for

the V_{th} shift observed experimentally in Fig. 2f. The subsequent positive scan suggested by the reviewer would indeed provide strong confirmation. In our simulation protocol, the comparison between Steps V and VII serves an equivalent purpose, as both sequences probe the asymmetry in the energy landscape induced by the oriented dipoles. The increased threshold for switching in the direction opposite to the previous polarization (Step VI) confirms that the system "remembers" its prior state, which is the essence of the SOM behavior.

The underlying mechanism for this behavior, as explained in the manuscript, is the dipole-orientation-dependent growth of the CF. The residual polarization from the initial forming process (Step III) creates a preferred direction. Switching in the same direction (Step V) benefits from this pre-existing polarization, resulting in a lower V_{th}. However, reorienting the dipoles to the opposite direction (Step VII) requires overcoming a higher energy barrier, leading to an increased V_{th}. This finding is consistent with the intuitive explanation of the asymmetry, in which directional conduction clusters exist (Ravsher et.al. IEDM 2021).

Therefore, we revise the relevant section in the manuscript to clearly state the observed asymmetry in V_{th} between Steps V and VII in **page 9**: "The polarity-dependent V_{th} shift can be exploited in the SOM application. The asymmetry arises from different dipole-orientation-dependent CF growth: States III and V share the same dipole orientations, and state VII exhibits opposite dipole orientation to state III. The residual polarization in state V reduces its V_{th} relative to state III, while reorienting dipoles to the opposite direction (state VII) requires a higher V_{th} to overcome the energy barrier."

Reviewer #2:

This manuscript reports on a combined computational and experimental investigation of the switching threshold mechanism in amorphous chalcogenides. This mechanism consists of an abrupt increase in conductivity at a critical threshold voltage and is exploited in the selector elements of phase-change random-access memories (PCRAMs). Moreover, it plays a key role in selector-only memories, which achieve faster switching speeds and lower power consumption than conventional phase-change memories, at the expense of losing non-volatility.

The physical mechanism responsible for threshold switching has been debated for several decades. In particular, both electronically driven and structurally driven models have been proposed. The main claim of the paper is that threshold switching in GeSe—a prototypical selector material—stems from dipolar ordering of atoms induced by the external electric field. This ordering, in turn, guides the formation of conductive filaments oriented along the electric field. The authors also argue that this mechanism can explain the threshold voltage asymmetry in selector-only memories as well. These claims are supported by atomic-resolution angstrom-beam electron diffraction and ab initio molecular dynamics simulations performed under an applied electric field.

The topic of the paper is certainly interesting and important, and the manuscript is well written. It has already undergone a first round of review. While the second reviewer provided a positive assessment, the first referee raised serious concerns about the validity of the simulations, noting that the bulk dipole moment can be computed within the Berry-phase approach only in systems with a finite electronic energy gap. I fully agree with this point. Apparently, this comment was triggered by an incorrect statement in the first version of the manuscript, namely that the ON state is a zero-gap state. The authors have clarified that, in fact, this state exhibits a non-negligible energy gap, and that the gap never closes during the ab initio simulations. I find their reply convincing, as they now provide computational evidence for the presence of a finite HOMO-LUMO gap in the new figures included in the revised manuscript and in the Supplementary Information. Nevertheless, the

authors should clarify the following points before I can recommend publication of this work in Nature Communications.

Reply: We sincerely thank the reviewer for their positive feedback on the clarity of the manuscript and acknowledge the novelty of this work.

Q1: I agree that monitoring the gap between the HOMO and LUMO states is essential to assess the validity of the Berry-phase approach. However, based on Figure 4c, one could argue that the "band gap"—or, more precisely, the (Tauc) optical gap—remains on the order of 0.5 eV throughout the simulation, and that the states appearing within this gap correspond to midgap or defect states. Typically, these states are strongly localized, hence I am somewhat puzzled by the fact that in your "ON" models they seem to be extended. The authors should comment on this point. The charge-density isosurfaces shown in Figures 4e–g do not provide sufficient evidence for delocalization, since one can always produce a delocalized isosurface by tuning the isovalue. Computing the IPR values is the correct approach. The authors did compute this quantity; however, it is not possible to compare the IPR values of these delocalized states with those of the other relevant states (including the defect state in the "OFF" model) in Figure 4c because the plots are too small. Even after zooming in, it is not clear whether the IPR value of the LUMO state in the "ON" model is significantly lower than that of the LUMO state in the "OFF" model. The authors should include additional plots of DOS and IPR versus energy (similar to Figure S10a) for several "OFF" and "ON" models in the main text to facilitate comparison between the IPR values.

Reply: We thank the reviewer for this insightful comment and for highlighting the importance of clearly distinguishing the nature of the gap states and providing unambiguous evidence for their delocalization. We agree that a thorough analysis is crucial. Below, we address the points raised and have supplemented the main text with new figures to facilitate a direct comparison.

The reviewer correctly points out that strongly localized mid-gap or defect states typically have a negligible impact on the optical gap. In our "ON" state models, which represent a fully formed conductive filament, the states appearing within the gap are indeed distinct from traditional localized defect states. As the reviewer suggested, the Inverse Participation Ratio (IPR) is the definitive metric for quantifying localization, with a low IPR value indicating a delocalized state.

To address the concern directly, we now include a detailed comparison of IPR values across different models in Fig. R4 of the revised manuscript (Fig. S8 in the SI). The data unequivocally shows that the HOMO and LUMO states in the "ON" models exhibit low IPR values (~0.01), comparable to those of the delocalized HOMO state in the "OFF" models. In contrast, a genuine defect state in a fast-quenched "OFF" model exhibits a much higher IPR value (0.032), confirming its localized character. This analysis supports our conclusion that the conductive filament in the "ON" state is characterized by delocalized HOMO and LUMO states.

We apologize for the lack of clarity in the original Figure 4c. In direct response to the reviewer's request, we have now created and included enhanced plots of the DOS and IPR versus energy for representative "OFF" and "ON" models (in Fig. S8 for the supporting information). These new plots are larger in scale and allow for a clear, visual comparison of the IPR values. They confirm that the IPR of the LUMO state in the "ON" model is indeed similar to that of the delocalized HOMO in the "OFF" model, and significantly lower than that of a localized defect state.

Changes made to the manuscript on page 11: "Supporting this distinction, the significantly lower inverse participation ratio (IPR) of the ON-state molecular orbitals (≈0.01, scaled by 2000 in Fig. 4e) compared to the localized defect state (0.032, Fig. 4d) confirms the delocalized electronic nature of the CF. A detailed IPR comparison of these states is provided in Fig. S8.".

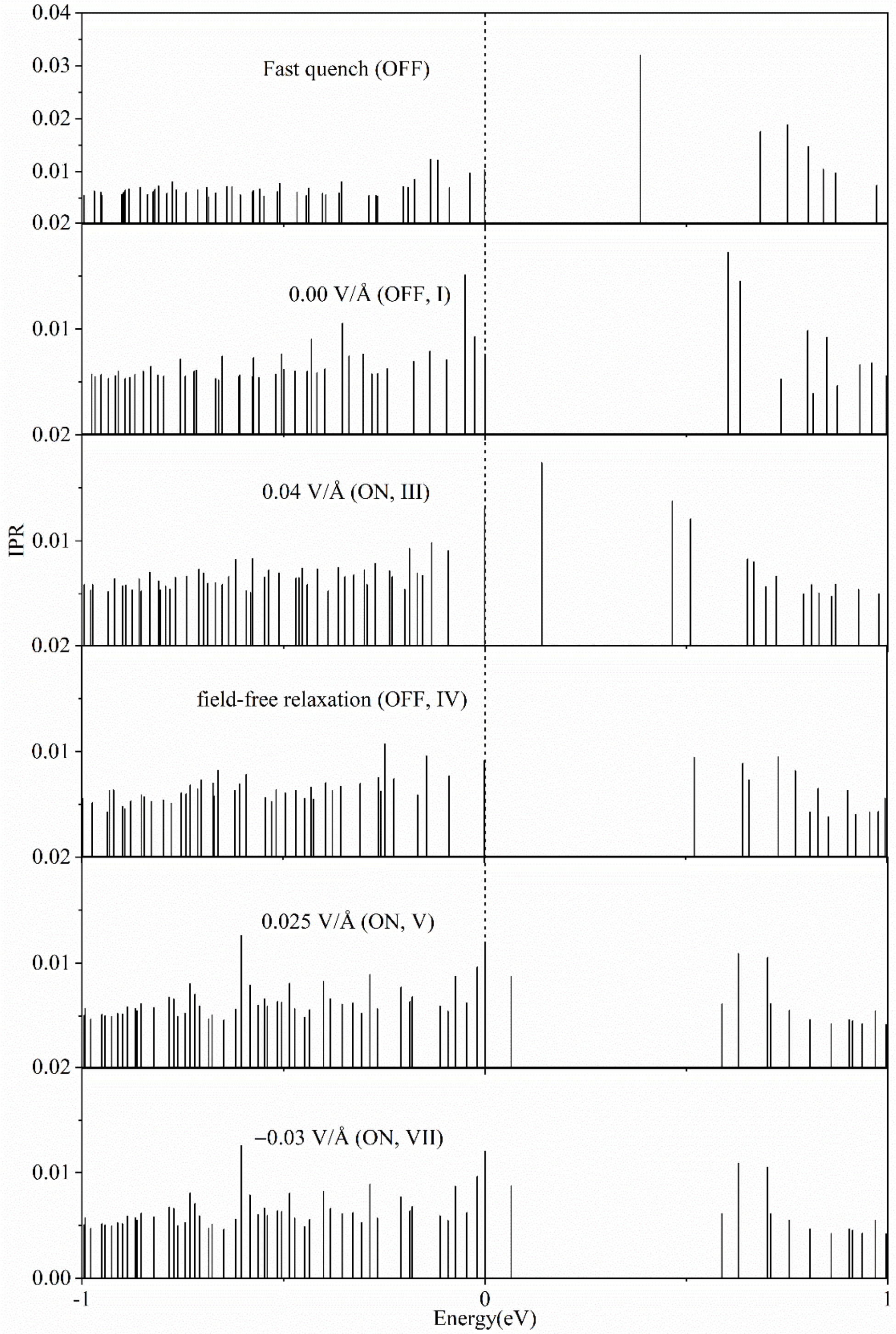

Fig. R4 (or Fig. S8 in SI) IPR values across the structural evolution sequence. The IPR was calculated for the key states in the sequence: the initial fast-quench, OFF (I), ON (III), OFF (IV), ON (V), to the ON (VII) models. The OFF (IV) model serves as the initial structure for the subsequent ON (V), OFF (VI), and ON (VII) states.

Q2: Furthermore, based on Figure S10 (which shows results for an electric field aligned along the x direction), one could argue that the LUMO state appears more localized than the states deep in the valence and conduction "bands," apparently contradicting the claims of the paper. Nevertheless, a relatively large IPR value in a small supercell does not necessarily imply strong localization in all directions; it could also reflect a quasi-1D or quasi-2D character of the electronic state (this point has been discussed in previous computational works on the electronic properties of PCMs).

Reply: We thank the reviewer for raising the important point regarding the interpretation of IPR values. We agree that this requires more careful consideration.

On the apparent contradiction regarding the LUMO state in Figure S10:

The reviewer correctly notes that, in Figure S11 (electric field along the x-direction), the LUMO state exhibits a slightly larger IPR value (0.01~0.015) than states deeper in the valence and conduction bands (~0.007). We appreciate the reviewer's careful examination. However, as the reviewer astutely points out, a relatively large IPR value in a small supercell does not necessarily imply strong localization in all directions. Crucially, both the HOMO and LUMO states, with an IPR of ~0.01, fall well within the established range for delocalized states according to the literature (Nat. Commun. 10, 3065 (2019)), which defines delocalized states as having IPR values between 0.01 and 0.02. States with IPR values ranging from approximately 0.03 to 0.06 are considered partially localized, whereas strongly localized defect states can exhibit IPR values of approximately 0.09 or higher. Therefore, the HOMO and LUMO states in Figure S11, along with the deeper band states, are all consistent with being delocalized. The slight difference in their IPR values reflects variations in the spatial distribution of the electronic wavefunction rather than a fundamental difference in their localized or delocalized character.

On the implications of IPR values in quasi-1D or quasi-2D systems:

We sincerely thank the reviewer for highlighting the nuanced interpretation of IPR values, particularly in systems with quasi-1D or quasi-2D character, where

geometric confinement plays a crucial role. We fully agree with this important perspective. A relatively large IPR value in such contexts need not imply strong, isotropic localization in the traditional sense. Instead, it can be a direct signature of a substantial spatial inhomogeneity in the electron density distribution along the confined dimension (e.g., along the quasi-1D chain). The IPR quantity essentially measures the inverse of the number of sites over which the wavefunction is significantly distributed. Within a structurally confined pathway, the electronic state can remain extended along that path but exhibit a non-uniform distribution, leading to an increased IPR. This distinguishes it from a state that is tightly localized to a very small number of sites.

To clarify our methodology, the IPR is calculated using the formula:

$$IPR_{nk} = \frac{\sum_a |c_{nka}|^4}{\left(\sum_a |c_{nka}|^2\right)^2}$$

, where *n* is the band index, *k* is the k-point index, *a* is the atom index, c_{nka} are the wavefunction coefficients. This metric essentially measures the inverse of the number of atoms over which the wavefunction is significantly distributed. A key insight is that the IPR value is intrinsically tied to the uniformity of the wavefunction distribution across its spatial extent, not merely the absolute size of an isosurface region.

For instance, if a molecular orbital (MO) is distributed over four atoms with equal coefficients (e.g., 0.25 each), the resulting IPR will be 1.85 times lower than if the same MO were distributed over the same four atoms but with uneven coefficients (e.g., 0.45, 0.05, 0.35, 0.15). In the latter case, indicating a less uniform distribution, the IPR value is higher. This result illustrates that a high IPR can indeed signify localization within a specific region, even if that region itself has a quasi-1D character. As the reviewer noted, and as supported by computational studies on PCMs, a quasi-1D filamentary path can exhibit a characteristically large IPR value because the electronic state is confined to that specific pathway. Thus, our observation of a moderately elevated IPR is consistent with the formation of a delocalized state channeling through a quasi-1D conductive filament, which aligns with the physical picture we are proposing.

The IPR values observed for the HOMO and LUMO states in our "ON" models are

indicative of delocalized states, consistent with established literature thresholds. To make this point clearer, we comment on the larger IPR value of LUMO than the deep states, as suggested by the reviewer; the larger LUMO here may reflect a quasi-1D or quasi-2D character of the electronic state, as in the conductive filament we proposed. In page 11, we add the following sentence: "The slightly larger IPR values of both the LUMO and HOMO, compared to the deep states, can be attributed to the quasi-one-dimensional (quasi-1D) character of the CF structure, analogous to the electronic states found in quasi-1D or quasi-2D phase-change materials."

Q3: Finally, I am wondering why the authors do not observe strongly localized states around Ge–Ge homopolar bonds or other defects in the ON models, but only in the fast-quenched OFF model. While I understand that fast-quenched models contain a higher concentration of Ge–Ge bonds, I would still expect such states to appear in at least some ON models. The authors should briefly comment on this point.

Reply: We thank the reviewer for this insightful question, which allows us to clarify a key distinction between the structural states generated by different preparation kinetics. The absence of strongly localized states in our "ON" models is primarily a consequence of two factors: 1) the significantly lower concentration of Ge–Ge homopolar bonds in the initial, experimentally relevant "OFF" state, and 2) the specific, minimally disruptive atomic rearrangement pathway during the switching process.

The initial "OFF" state has an inherently low defect concentration.

As the reviewer correctly notes, the concentration of Ge–Ge homopolar bonds is highly dependent on the quenching rate. Our "ON" models are generated from an initial "OFF" state that was created using a slow quenching rate (10 K/ps), which is much closer to experimental conditions (approximately 1000 K/ns) than the fast-quenched model (50 K/ps). As shown in Fig. R5, this slow-quenched initial model contains only about 10 Ge–Ge bonds in a 200-atom system, with no Se–Se homopolar bonds. This number is substantially lower than the homopolar bond concentration found in a comparable amorphous $Ge_2Sb_2Te_5$ system. Therefore, the underlying "raw

material" for the switching event in our simulations has a very low density of the specific defects that lead to strong localization.

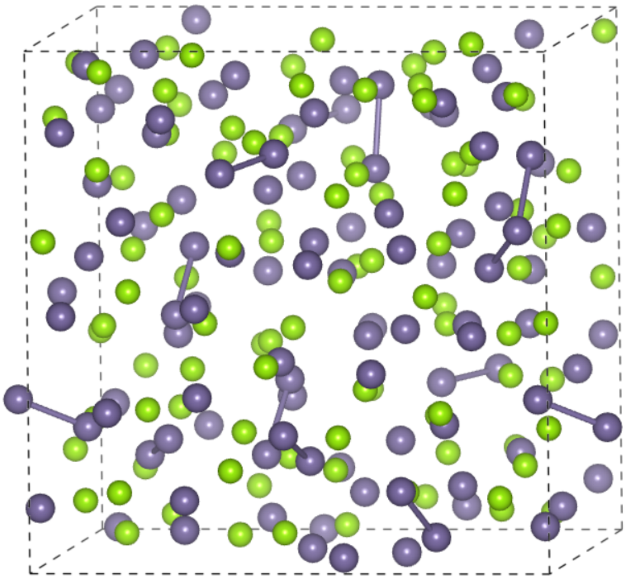

Fig. R5 The atomic structure of the initial amorphous GeSe model using the slowly quenching rate (10K/ps). Using the cutoff of 3.0 Å, there are a few Ge–Ge bonds and no Se–Se homopolar bonds.

The switching mechanism does not favor the creation or activation of new localized states around Ge–Ge homopolar bonds.

The process of switching to the "ON" state is characterized by a collective and relatively gentle atomic rearrangement within the incipient filamentary region, with an average atomic displacement of only about 0.2 Å. This minimal movement is sufficient to induce a significant change in the electronic structure, introducing a new, delocalized state near the HOMO that defines the conductive channel. Crucially, the pre-existing Ge–Ge bonds in the initial matrix do not undergo a structural change that would convert them into strongly localized mid-gap states.

More fundamentally, for a Ge–Ge bond to become part of a conductive filament, it would require a long-range, directed diffusion of Ge atoms to a specific channel location, a process that is not only slow but also lacks a clear driving force in our modeled mechanism. There is no additional force in our simulations that would guide randomly oriented Ge atoms from various directions to coalesce into a single direction. Thus, the formation mechanism we observe naturally leads to a delocalized electronic

state intrinsic to the filament structure, rather than activating localized states from pre-existing point defects.

Therefore, the combination of a high-quality (low-defect) initial state and a switching mechanism involving minimal, collective atomic motion explains why strongly localized states are a hallmark of the highly disordered, fast-quenched "OFF" model but are not observed in the well-defined, metastable "ON" states. To briefly comment on this point, we add a sentence on page 11: "The ON states (III, V, VII) exhibit no obvious localized states around the limited number of Ge–Ge bonds, as evidenced by the slower quenching rate and a minor atomic displacement of ~0.2 Å during the switching process.".

Reviewer #3:

This manuscript presents a groundbreaking study that successfully resolves a long-standing challenge in the field of amorphous chalcogenides: the atomic-scale mechanism of threshold switching (TS). By synergistically combining state-of-the-art experimental techniques (Angstrom-Beam Electron Diffraction, ABED) and advanced computational methods (Electric-Field-coupled Ab Initio Molecular Dynamics, EF-MD), the authors provide compelling evidence for a field-driven dipolar ordering paradigm. This work shifts the understanding of TS from purely electronic or slow structural models to a unified structural-electronic mechanism governed by ultrafast, non-Arrhenius dipolar reconfiguration. The findings are of fundamental importance and have significant implications for the design of next-generation ultrafast, low-power memory technologies.

The primary weaknesses raised by the previous reviewers, concerning the computational framework's validity and the clarity of experimental methods, have been extensively and successfully addressed in the revision and rebuttal. The added methodological details and supplementary data significantly strengthen the manuscript. While the 3600-atom model is impressive and shown to be representative, it still represents a simplified, defect-free region of the amorphous

material. Real devices may contain a broader distribution of intrinsic defects and stoichiometric variations that could influence the statistical formation of filaments. The authors acknowledge this through their discussion of stochastic branching at topological defects. The proposed mechanism is meticulously demonstrated for amorphous GeSe. While it is highly plausible that similar dipolar effects exist in other chalcogenides (e.g., Ge-Sb-Te, As-Se systems), direct experimental and computational evidence for other compositions is outside the scope of this paper. The generality of the paradigm remains to be fully tested across the material class.

The dielectric spectroscopy (Fig. 5) shows a significant dipole polarization response. Could the authors comment on how the measured relaxation times of these dipoles relate to the picosecond switching times observed in EF-MD? Is the measured response primarily from the bulk matrix, while the switching involves a more correlated, sub-ensemble of "active" dipoles?

In the hierarchical CF growth model, the dipolar chains form first, and the CF grows perpendicularly. What is the precise electronic structure at the interface between a dipolar chain and the nascent CF? Is the chain itself conductive, or does it merely create a localized strain or potential field that guides the nucleation of a metallic filament?

Finally, this manuscript represents a seminal contribution to the fields of phase-change materials, non-volatile memory, and the physics of disordered systems. The work is exceptionally well-executed, the evidence is robust and multi-faceted, and the findings are of the highest significance. The authors have satisfactorily resolved all major criticisms. I strongly recommend acceptance of this manuscript for publication after minor revision.

Reply: We are very grateful for the exceptionally positive and insightful evaluation of our work. The reviewer encouraged us greatly by describing our work as a “groundbreaking study” that “successfully resolves a long-standing challenge” and represents a “seminal contribution”. We are thrilled that the significance of our proposed paradigm shift, from purely electronic models to a unified structural-

electronic mechanism, resonated so strongly with the reviewer. The supportive comments and the remaining nuanced questions provide a perfect opportunity to clarify further and strengthen the narrative of the manuscript.

Q1: The dielectric spectroscopy (Fig. 5) shows a significant dipole polarization response. Could the authors comment on how the measured relaxation times of these dipoles relate to the picosecond switching times observed in EF-MD? Is the measured response primarily from the bulk matrix, while the switching involves a more correlated, sub-ensemble of "active" dipoles?

Reply: We sincerely thank the reviewer for this insightful question, which allows us to clarify the relationship between the different dipole dynamics observed at various time scales and under different driving conditions. The reviewer's understanding is indeed precise, and we agree that the dipole response measured by conventional dielectric spectroscopy (DS) and the ultrafast switching observed in our electric-field molecular dynamics (EF-MD) simulations originate from distinct yet related physical mechanisms.

The dielectric relaxation observed in the MHz range (corresponding to a characteristic time on the order of microseconds) under a weak AC field (as shown in Fig. 5) primarily reflects the thermally activated reorientation or fluctuation of dipoles within the bulk matrix of the material under near-equilibrium conditions, which is under a very weak electric field. This is a linear response measurement that probes the average behavior of the entire dipole population. The relaxation time in this context is governed by the energy barriers to dipole rotation in the amorphous structure, which is typically slow.

In contrast, the picosecond-scale switching dynamics revealed by our EF-MD simulations occur under an ultra-high, non-equilibrium electric field. This process does not represent the slow, thermally activated flipping of individual dipoles. Instead, it corresponds to a field-driven, collective reconfiguration of a strongly correlated sub-ensemble of "active" dipoles. This cooperative motion leads to the rapid formation of a percolating dipolar chain or filament, which is the core mechanism of the threshold

switching we propose. The characteristic frequency of this process falls into the THz range, consistent with ultrafast atomic motions and electronic polarization changes captured by AIMD.

The MHz dielectric relaxation and the pico-second switching time describe different phenomena: the former is the thermally averaged, linear response of the bulk, while the latter is the nonlinear, collective response of a privileged sub-ensemble under a strong field. They are complementary, with the fast switching causing the macroscopic conductance change, and slow relaxation reflecting the background dipole mobility that enables it. To make this point clear, we modify the manuscript and add the following sentence: "The slower dipole relaxation time measured experimentally, compared to the picosecond switching time observed in EF-MD simulations, results from the very weak electric field applied, which confines the measurement to the linear response regime and thus probes the average behavior of the entire dipole population."

Q2: In the hierarchical CF growth model, the dipolar chains form first, and the CF grows perpendicularly. What is the precise electronic structure at the interface between a dipolar chain and the nascent CF? Is the chain itself conductive, or does it merely create a localized strain or potential field that guides the nucleation of a metallic filament?

Reply: We thank the reviewer for this insightful question regarding the electronic structure at the critical interface between the initial dipolar chain and the nascent conductive filament (CF). The reviewer's intuition is highly relevant. Our analysis indicates that the dipolar chain primarily serves as a template that generates a localized strain and electrostatic potential field, which in turn guides the nucleation and growth of a conductive filament.

The formation of the dipolar chain introduces significant structural frustration at its boundary with the surrounding disordered matrix. This frustration manifests as bond distortion and coordination defects, such as under-coordinated Ge^{3+} species, as highlighted in Fig. S15. This structural frustration, creating local strain, generates

delocalized electronic states near the Fermi level, enabling high conductivity through CFs percolating across the material.

High conductivity emerges only when the CF grows perpendicularly, forming a continuous, percolating pathway. The electronic states near the Fermi level that are responsible for conduction are thus delocalized states intrinsic to the mature CF structure, not the precursor dipolar chain. Therefore, the dipolar chain's primary role is to create a favorable local environment (through strain and potential fields) that directs the growth of a conductive pathway, which then develops its own high conductive character.

To clarify this hierarchical mechanism in the manuscript, we have added a brief sentence on **page 11** in the section describing Fig. 4e: "The formation of CF in Fig. 4e is driven by the field-favored Ge–Se chain (Fig. 3e), which serves a structural template.".

Reviewer #1:

I appreciate the authors' comprehensive efforts in demonstrating the complete "OFF-ON-OFF-ON" switching cycle via field-free relaxation, which confirms the recovery of the insulating state. The computational validation of threshold voltage asymmetry now provides a physical explanation for the state-dependent memory behavior observed in SOM devices. I feel that these revisions successfully bridge the gap between atomistic simulations and experimental observations by establishing a unified paradigm for field-driven dipolar ordering in amorphous chalcogenides. I am satisfied with the technical clarifications provided.

Reply: Thank you for your positive feedback. We greatly appreciate your recognition that our revisions, particularly the demonstration of the complete switching cycle and the computational validation of threshold voltage asymmetry, have successfully bridged the gap between atomistic simulations and experimental observations. We are pleased that you find the technical clarifications satisfactory and that the established paradigm for field-driven dipolar ordering helps unify the interpretation. We believe these additions have strengthened the manuscript, and we thank you again for your insightful comments throughout the review process.

Upon the request from the Editor, I have also examined the responses to the Reviewer #3's comments. The first question on the relation between relaxation time and switching time was answered satisfactorily and the modification to the manuscript is also reasonable. While the rebuttal to the second comment is reasonable, the manuscript modification is rather short. The Reviewer questioned on the precise electronic structure at the interface between the dipolar chain and the nascent CF, specifically asking whether the chain itself is conductive or merely provides a localized strain/potential field. In the rebuttal letter, the authors provided a highly convincing physical explanation, but the single sentence added to the revised manuscript is too brief. I recommend that the authors incorporate a concise version of their rebuttal explanation directly into the main text. Once this minor addition is made, I believe the manuscript will be fully ready for publication.

Reply: Thank you for your careful evaluation of our responses and for the constructive suggestion. We agree that incorporating a more detailed physical explanation of the dipolar chain's role into the main text would enhance the manuscript's clarity.

We make the corresponding changes on **page 8** by adding following sentences. "The dipolar chain primarily serves as a structural template, which induces a localized strain and electrostatic potential field. This field promotes bond distortion and coordination defects (e.g., under-coordinated Ge^{3+}, as highlighted in Supplementary Figure 15a) at the chain-matrix interface. These structural perturbations create delocalized electronic states near the Fermi level, thereby guiding the nucleation and subsequent growth of a conductive filament. The resultant high conductivity is thus an intrinsic property of the matured, percolating CF, rather than of the precursor chain itself."

Reviewer #2:

The authors have addressed my comments and concerns in a satisfactory way, and I recommend publication of the manuscript in its present form.

I only have one final remark. In their response, the authors state that "Crucially, both the HOMO and LUMO states, with an IPR of ~0.01, fall well within the established range for delocalized states according to the literature (Nat. Commun. 10, 3065 (2019)), which defines delocalized states as having IPR values between 0.01 and 0.02."

However, the range of IPR values associated with delocalized states depends on the system size: the larger the system, the smaller the IPR of extended states. In the thermodynamic limit, the IPR of a delocalized state vanishes. Since the system size considered in Nat. Commun. 10, 3065 (2019) differs from that of the models used here, the range of values reported in that study cannot be used to discriminate between localized and delocalized states in this work.

Reply: Thank you for this important and clarifying comment regarding the system-size dependence of the IPR values. You are absolutely correct that the IPR of a truly

delocalized state scales with system size and vanishes in the thermodynamic limit. Therefore, the absolute numerical range from a reference with a different model size cannot be directly used as a universal threshold.

We have followed your suggestion and revised the manuscript accordingly. The relevant sentence now includes a note clarifying this dependence, ensuring that the values from the reference (Nat. Commun. 10, 3065) are presented strictly for contextual comparison rather than as a direct criterion. The revised text reads:

"This is consistent with the reference[38], in which IPR values of 0.01–0.02 (0.03–0.06) were reported for (partially) delocalized states; it should be noted, however, that the larger system size in that study results in generally lower IPR ranges."

We believe this addition appropriately addresses the point and improves the precision of our discussion. Thank you again for your careful reading and valuable insight, which has strengthened the final manuscript.
